# LCMS-Metabolomic Profiling and Genome Mining of *Delftia lacustris* DSM 21246 Revealed Lipophilic Delftibactin Metallophores

**DOI:** 10.1021/acs.jnatprod.4c00049

**Published:** 2024-05-13

**Authors:** Mohammed M. A. Ahmed, Paul D. Boudreau

**Affiliations:** † Boudreau Lab, Department of BioMolecular Science, School of Pharmacy, University of Mississippi, University, Mississippi 38677, United States; ‡ Department of Pharmacognosy, Al-Azhar University, Cairo 11651, Egypt

## Abstract

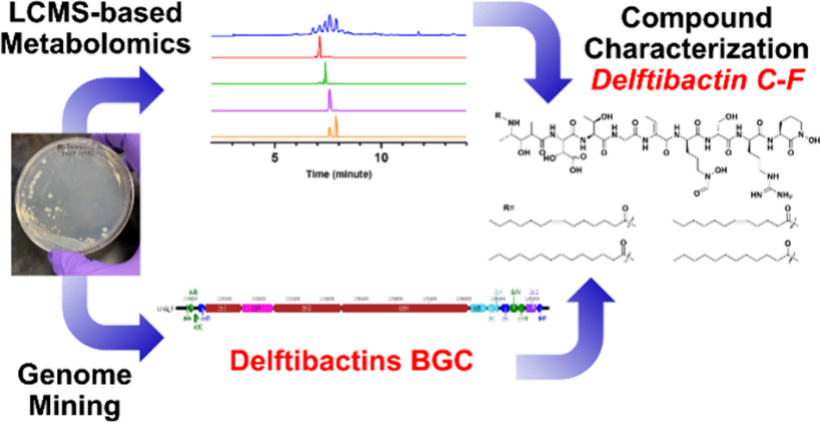

Bacteria have evolved various strategies to combat heavy metal stress, including the secretion of small molecules, known as metallophores. These molecules hold a potential role in the mitigation of toxic metal contamination from the environment (bioremediation). Herein, we employed combined comparative metabolomic and genomic analyses to study the metallophores excreted by *Delftia lacustris* DSM 21246. LCMS-metabolomic analysis of this bacterium cultured under iron limitation led to a suite of lipophilic metallophores exclusively secreted in response to iron starvation. Additionally, we conducted genome sequencing of the DSM 21246 strain using nanopore sequencing technology and employed antiSMASH to mine the genome, leading to the identification of a biosynthetic gene cluster (BGC) matching the known BGC responsible for delftibactin A production. The isolated suite of amphiphilic metallophores, termed delftibactins C–F (**1**–**4**), was characterized using various chromatographic, spectroscopic, and bioinformatic techniques. The planar structure of these compounds was elucidated through 1D and 2D NMR analyses, as well as LCMS/MS-based fragmentation studies. Notably, their structures differed from previously known delftibactins due to the presence of a lipid tail. Marfey’s and bioinformatic analyses were employed to determine the absolute configuration of the peptide scaffold. Delftibactin A, a previously identified metallophore, has exhibited a gold biomineralizing property; compound **1** was tested for and also demonstrated this property.

Heavy metal pollution in the environment is a major concern due to its negative consequences on ecological systems as well as human health.^1−4^ Bacteria require some heavy metals as micronutrients to maintain their biological processes and metabolic functions.^5^ However, these metals can also inhibit the same bacteria when elevated to toxic levels.^6^ Other heavy metals such as mercury, cadmium, and lead have no reported biological role and are very toxic, even in very low concentrations.^7^ In response to the challenges posed by heavy metal stressors, bacteria have evolved a range of effective mechanisms to counteract metal toxicity.^8−14^ One prominent strategy involves the secretion of small-molecule metal chelators known as metallophores.^9−12^ These compounds possess the ability to bind, sequester, or neutralize hazardous heavy metals.^11,12^ Furthermore, several studies have been conducted to unveil the potential of bacterial metallophores in the bioremediation of heavy metals from the environment.^11,12,15^

Hofmann and colleagues have reviewed the potential of metallophores in the bioremediation of heavy metals.^16^ Notably, these metabolites exhibit structural features that facilitate their affinity for metal binding, often with a degree of selectivity toward particular metals.^17^ Metallophores can also play multiple roles in toxic metals. For instance, yersiniabactin, a bacterial siderophore, plays a role in mitigating copper stress triggered by the human host to protect against infection by forming a stable complex upon binding with copper.^18−20^ This distinctive property of yersiniabactin has sparked interest in its potential use for copper remediation.^21^ It has also been investigated for the removal and retrieval of other metals like nickel and magnesium from wastewater.^22^ Delftibactin A, a bacterial metallophore, was discovered by Johnston and co-workers.^11^ This small molecule, isolated from a strain of *Delftia acidovorans*, allows the bacterium to survive metal stress from ionic gold.^11^ Delftibactin A exhibits the capability to bind and transform soluble toxic gold ions into inert metallic gold nanoparticles, rendering them nontoxic to the bacterium.^11,23^ This metallophore possesses a scaffold characterized by a hybrid polyketide synthase–nonribosomal peptide synthetases (PKS-NRPS) pathway, featuring hydroxamate and carboxylate functionalities that contribute to its metal-binding properties.^11^

In this study, we unveil the discovery of a suite of delftibactin lipopeptide metallophores through the application of comparative metabolomics and genomics. The isolated compounds exhibit the conserved peptide backbone of delftibactin A, but our compounds also possess distinct lipid tails characterized by varying lengths and degrees of saturation not reported in the original isolation of the delftibactins.^11^ Furthermore, we tested the interaction between compound **1** and various metals, specifically gold, copper, and iron. The results revealed distinct interactions between compound **1** and each metal. Importantly, the tested compound (**1**) still possesses the previously reported gold biomineralizing capability of delftibactin A.^11^

## Results and Discussion

### Comparative Metabolomic Profiling of *Delftia lacustris* under Iron Limitation

**Figure 1:**
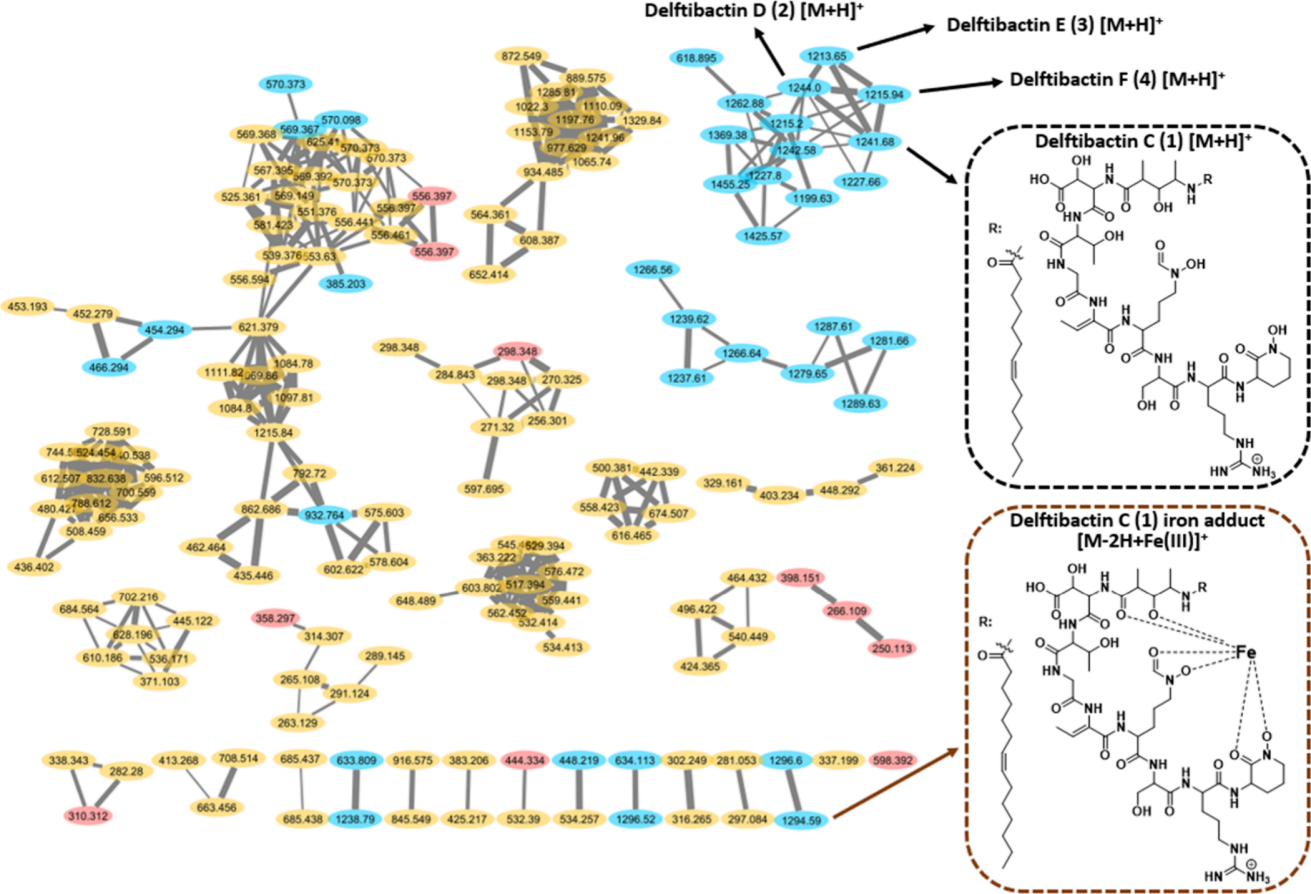
Metabolomic analysis of *D. lacustris* culture supernatants under low and high iron conditions using molecular networking. This analysis revealed clusters of putative delftibactin analogs (e.g., delftibactin C: [M + H]^+^ at 1241 *m*/*z* boxed in black, [M – 2H + Fe]^+^ at 1294 *m*/*z* boxed in brown). Nodes are colored by the culture conditions in which they were observed: blue = low iron, red = high iron, and yellow = both. Thicker lines indicate higher cosine scores.

To investigate the secretion of siderophores by *D. lacustris*, we followed a methodology we developed using comparative metabolomics with and without excess iron.^24^ In essence, we cultivated the bacteria in an iron-limiting medium with and without iron supplementation. We then processed the culture supernatants by RP-SPE with C18 and tested the middle polarity fractions (50% aqueous MeCN) using LCMS to identify the unique metabolites under each circumstance. LCMS revealed four major eluting ions corresponding to four compounds (**1**–**4**) that are produced only under iron limitation; these ions had *m*/*z* values of 1213.6414 [M + H]^+^, 1215.6579 [M + H]^+^, 1241.6730 [M + H]^+^, and 1243.6892 [M + H]^+^ (Figure S1, see Supporting Information). MS data were further analyzed using the GNPS platform^25^ and visualized with Cytoscape^26^ to see if these masses clustered together. Molecular networking showed these four precursor ions clustered together with many masses unique to the iron-limited condition (Figure 1). The results warranted further investigation of the MS/MS fragmentation patterns to assign putative structures associated with this cluster.

### MS/MS Fragmentation Spectra Annotation to Identify Putative Structures of Delftibactin A Analogs

Based on the antiSMASH^27^ genome mining of our *D. lacustris* genome, which identified the delftibactin A biosynthetic gene cluster in this organism (see below), we hypothesized that the unique cluster observed solely under the low iron-limited condition was analogs of delftibactin A (a known metallophore).^11^ To test this hypothesis, we used the delftibactin A fragmentation pattern as a template and investigated the MS/MS fragmentation spectra for the main ions discussed above. Not surprisingly, fragments derived from delftibactin A (such as fragments with *m*/*z* values of 904.4119, 773.3900, 672.3424, 615.3209, 532.2838, and 374.2152) were observed in the fragmentation spectra of those four major metabolites (**1**–**4**, see SI Figures S3, S4, S11, S12, S17, S18, S25, and S26). Nevertheless, compounds **1**–**4** exhibited distinct precursor ions with *m*/*z* values of 1213.6414 [M + H]^+^, 1215.6579 [M + H]^+^, 1241.6730 [M + H]^+^, and 1243.6892 [M + H]^+^. The comparison of the precursor ions for compounds **1** and **3**, as well as compounds **2** and **4**, revealed a mass difference of 28 Da. Meanwhile, a 2 Da difference separated compounds **1** and **2**, as well as compounds **3** and **4**. These findings led us to formulate the hypothesis that these compounds (**1**–**4**) share a delftibactin A-derived peptide backbone distinguished by the length and degree of saturation of an attached lipid tail. Careful annotation of the fragmentation mass spectrum for each compound revealed unique fragments bearing a lipid tail. We identified the lipid tails by calculating the mass differences between delftibactin A (calcd 1033.4914, [M + H]^+^) and our observed precursor masses. The observed mass differences of 208.1816 and 210.1978 Da suggested the presence of a 14-carbon fatty acid tail (a tetradecanoyl moiety) with and without a double bond for compounds **1** and **2**, respectively. Additionally, compounds **3** and **4** exhibited a 28 Da smaller lipid component (180.1499 and 182.1665 Da difference) when compared to those of compounds **1** and **2**. Consequently, it was postulated that compounds **3** and **4** possessed 12-carbon fatty acid tails (a dodecanoyl moiety) with or without a double bond, respectively. Prior studies have also reported amphiphilic siderophores with similar lipid tails.^28,29^ Our detailed analysis of the unique fragments in the MS/MS spectra of **1**–**4** was consistent with the biosynthetic incorporation of the lipid tail at the terminal NH_2_ of the 4-amino-3-hydroxy-2-methylpentanoic acid (Ahmpa) moiety (see Figures S3, S4, S11, S12, S17, S18, S25, and S26 for a comprehensive annotation of MS/MS fragments for **1**–**4**).

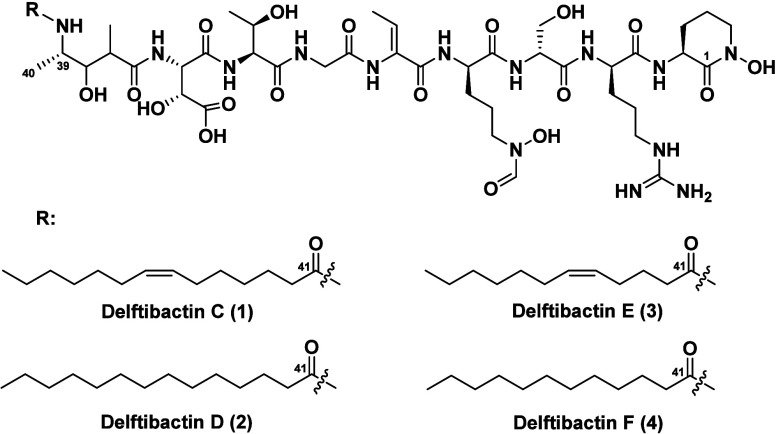



### Isolation and Structural Characterization of Delftibactins C–F

#### Isolation

Bacteria were grown in a defined medium for siderophores (DMS) extracted with Diaion HP20 resin and subjected to purification using RP-SPE cartridges followed by two routes of RP-HPLC to obtain pure compounds **1** (30 mg), **2** (11 mg), **3** (3 mg), and **4** (7 mg).

#### Planar Structure Elucidation

After conducting the initial MS/MS fragmentation analysis, we postulated that the isolated metabolites (**1**–**4**) were analogs of delftibactin A. To test this, we performed 1D- and 2D-NMR analysis and compared the obtained data to previously reported data of delftibactin A.^11,30^ Delftibactin C (**1**) was obtained as a yellowish white powder, and its molecular formula was determined as C_54_H_92_N_14_O_19_ based on positive HRESIMS *m*/*z* 1241.6730 [M + H]^+^ (calculated 1241.6736, – 0.5 ppm), indicating 17 degrees of unsaturation. Analysis of ^1^H NMR and ^13^C NMR (DEPT-Q) spectra of **1** (Table 1) showed the presence of 10 carbonyl signals, comprising 10 amide carbons at δ_C_ 166.9, 173.8, 172.9, 175.2, 167.8, 172.6, 174.0, 172.4, 178.2, and 175.3 and one carboxylic acid carbonyl at 175.3. Furthermore, an imine carbon at 158.5 ppm, along with a formyl singlet for each rotamer at δ_H_ 8.30 and 7.96 (δ_C_ 164.1 and 159.7), was also detected. Through examination of cross peaks in TOCSY, it was determined that compound **1** has nine distinct spin systems. Although the 1D and 2D NMR data exhibited a strong agreement with those of delftibactin A, there were also unique peaks detected within the NMR data of **1** (Figures S5–S9). In agreement with our MS/MS annotation of compound **1** (Figures S3 and S4), the lipid backbone showed 10 methylene signals at δ_C_ 37.4 (δ_H_ 2.19, t, *J* = 7.5), 27.0 (1.62, m), 29.9 (1.32, m), 30.0 (1.33, m), 28.0 (2.06, m), 28.1 (2.04, m), 30.8 (1.35, m), 30.6 (1.33, m), 32.9 (1.30, m), and 23.7 (1.32, m). Additionally, two olefinic signals were observed at 130.6 (5.35, m) and 131.0 (5.35, m) along with a methyl signal at 14.5 (0.90, t, 7.0) (Table 1). The attachment of the lipid tail to the delftibactin backbone was established based on the observed HMBC cross peaks between the methine proton (H-39) of the Ahmpa moiety at δ_H_ 3.96 and the fatty acid carbonyl (C-41, δ_C_ 175.3) (Figures S8 and S30). The location of the double bond in the lipid tail was established as being between the seventh and eighth carbon atoms of the lipid (C-47 and C-48), based on the HMBC data (Figures S8 and S30). Notably, HMBC cross peaks were observed from the methylene protons (H-42, δ_H_ 2.19) to the carbonyl (C-41, δ_C_ 175.3) and to two methylene carbons (C-43, δ_C_ 27.0 and C-44, δ_C_ 29.9); from methylene protons (H-43, δ_H_ 1.62) to two methylene carbons, C-44 (δ_C_ 29.9) and C-45 (δ_C_ 30.0); and from H-46 (δ_H_ 2.06) to both methylene C-45 and double bond C-48 (δ_C_ 130.6). This information, combined with the tandem mass spectrometry assignments, led to the identification of compound **1** as a 7-tetradecenoic acid analogue of delftibactin A.

**Table 1:** ^1^H (500 MHz) and ^13^C (125 MHz) NMR Chemical Shifts of **1**–**4** in CD_3_OD

		**1**		**2**		**3**		**4**	
residue	position	δ_C,_ type	δ_H_ (*J* in Hz)	δ_C,_ type	δ_H_ (*J* in Hz)	δ_C,_ type	δ_H_ (*J* in Hz)	δ_C,_ type	δ_H_ (*J* in Hz)
Cyclic, N–OH-Orn	1	166.9, C		166.9, C		167.0, C		166.9, C	
	2	52.6, CH_2_	3.67, m, 3.62, m	52.6, CH_2_	3.65, m, 3.61, m	52.6, CH_2_	3.66, m, 3.61, m	52.6, CH_2_	3.67, m, 3.62, m
	3	21.6, CH_2_	2.08, m, 1.97, m	21.6, CH_2_	2.09, m, 1.97, m	21.6, CH_2_	2.09, m, 1.97, m	21.6, CH_2_	2.09, m, 1.97, m
	4	28.5, CH_2_	2.04, m, 1.86, m	28.5, CH_2_	2.02, m, 1.88, m	28.5, CH_2_	2.06, m, 1.88, m	28.5, CH_2_	2.04, m, 1.87, m
	5	51.3, CH	4.51, m	51.3, CH	4.51, m	51.3, CH	4.51, m	51.3, CH	4.52, m
Arg	6	173.8, C		173.8, C		173.8, C		173.8, C	
	7	54.8	4.37, m	54.9	4.35, m	54.9	4.35, m	54.9	4.36, m
	8	29.6, CH_2_	1.98, m, 1.76, m	29.6, CH_2_	1.95, m, 1.75, m	29.6, CH_2_	1.96, m, 1.75, m	29.6, CH_2_	1.95, m, 1.75, m
	9	26.2, CH_2_	1.63, m	26.2, CH_2_	1.63, m	26.2, CH_2_	1.66, m	26.2, CH_2_	1.63, m
	10	41.9, CH_2_	3.17, m	42.0, CH_2_	3.16, m	42.0, CH_2_	3.16, m	42.0, CH_2_	3.17, m
	11	158.5		158.6		158.6		158.6	
Ser	12	172.9, C		173.0, C		173.0, C		173.0, C	
	13	57.9, CH	4.40, m	58.0, CH	4.38, m	57.9, CH	4.38, m	58.0, CH	4.38, m
	14	62.6, CH_2_	3.93, m, 3.86, m	62.6, CH_2_	3.91, m, 3.85, m	62.6, CH_2_	3.93, m, 3.86, m	62.6, CH_2_	3.93, m, 3.86, m
*N*^δ^-OH-*N*^δ^-formyl-Orn	15	175.2, C		175.4, C		175.2, C		175.3, C	
	16	56.3, 56.2, CH	4.33, m	56.4, 56.3, CH	4.31, m	56.3, 56.5, CH	4.30, m	56.3, 56.2, CH	4.30, m
	17	29.6, 28.9, CH_2_	1.95, m, 1.76, m	29.6, 28.9, CH_2_	1.98, m	29.6, CH_2_	1.95, m, 1.75, m	29.6, 28.8, CH_2_	1.95, m, 1.75, m
	18	24.8, 24.3, CH_2_	1.84, m, 1.75, m	24.8, 24.4, CH_2_	1.78, m	24.8, 24.1, CH_2_	1.86, m, 1.75, m	24.8, 24.4, CH_2_	1.78, m, 1.75, m
	19	47.0, 50.8, CH_2_	3.62, m, 3.57, m	47.0, 50.8, CH_2_	3.62, 3.56, m	47.0, 50.8, CH_2_	3.62, m	47.0, 50.8, CH_2_	3.63, m
	20	164.1, 159.7, CH	8.30, s, 7.96, s	164.2, 159.7, CH	8.30, s, 7.96, s	164.2, 159.7, CH	8.30, s, 7.96, s	164.2, 159.7, CH	8.31, s, 7.97, s
Dhb	21	167.8, C		167.9, C		167.9, C		167.7, C	
	22	131.9, C		130.9, C		130.9, C		130.9, C	
	23	132.3, CH	6.56, m	132.3, CH	6.56, m	132.4, CH	6.56, m	132.4, CH	6.57, m
	24	13.3, CH_3_	1.79, m	13.2, CH_3_	1.79, m	13.2, CH_3_	1.80, m	13.2, CH_3_	1.80, m
Gly	25	172.6, C		172.5, C		172.6, C		172.7, C	
	26	44.0, CH_2_	4.06, m, 4.00, m	44.2, CH_2_	4.05, m, 4.01, m	44.0, CH_2_	4.05, m, 4.00, m	44.2, CH_2_	4.05, m, 4.01, m
Thr	27	174.0, C		174.2, C		174.0, C		174.2, C	
	28	60.9, CH	4.30, m	60.9, CH	4.29, m	60.9, CH	4.30, m	60.8, CH	4.29, m
	29	67.8, CH	4.41, m	67.8, CH	4.42, m	67.8, CH	4.42, m	67.9, CH	4.42, m
	30	20.5, CH_3_	1.24, d (6.4)	20.5, CH_3_	1.25, d (6.5)	20.5, CH_3_	1.25, d (6.6)	20.5, CH_3_	1.25, d (6.6)
l-*erythro*-β-OH-Asp	31	172.4, C		172.5, C		172.9, C		172.5, C	
	32	57.9, CH	4.89, m	58.0, CH	4.87, m	58.0, CH	4.89, m	58.1, CH	4.87, m
	33	74.2, CH	4.34, m	74.2, CH	4.32, m	74.3, CH	4.33, m	74.2, CH	4.33, m
	34	175.3, C		175.3, C		174.8, C		174.8, C	
Ahmpa	35	178.2, C		178.3, C		178.2, C		178.2, C	
	36	44.3, CH	2.59, m	44.3, CH	2.58, m	44.3, CH	2.56, m	44.5, CH	2.58, m
	37	12.5	1.20, d (6.9)	12.5	1.20, d (6.9)	12.5	1.20, d (6.8)	12.5	1.20, d (6.7)
	38	75.9, CH	3.71, m	75.9, CH	3.71, m	75.9, CH	3.72, m	76.0, CH	3.71, m
	39	48.2, CH	3.96, m	48.2, CH	3.97, m	48.3, CH	3.97, m	48.2, CH	3.97, m
	40	16.7, CH_3_	1.17, d (6.6)	16.7, CH_3_	1.18, d (6.6)	16.8, CH_3_	1.18, d (6.8)	16.8, CH_3_	1.18, d (6.6)
fatty acid tail	41	175.3, C		175.4, C		175.3, C		175.4, C	
	42	37.2, CH_2_	2.19, t (7.5)	37.3, CH_2_	2.18, t (7.5)	36.8, CH_2_	2.20, t (7.5)	37.3, CH_2_	2.19, t (7.5)
	43	27.0, CH_2_	1.62, m	27.1, CH_2_	1.61, m	27.1, CH_2_	1.65, m	27.1, CH_2_	1.62, m
	44	29.9, CH_2_	1.32, m	30.3, CH_2_	1.29–1.32, m	27.8, CH_2_	2.08, m	30.1, CH_2_	1.29–1.32, m
	45	30.0, CH_2_	1.33, m	30.5, CH_2_	1.29–1.32, m	129.8, CH	5.36, m	30.5, CH_2_	1.29–1.32, m
	46	28.0, CH_2_	2.06, m	30.7^a^, CH_2_	1.29–1.32, m	131.8, CH	5.40, m	30.7^a^, CH_2_	1.29–1.32, m
	47	130.6, CH	5.35, m	30.7^a^, CH_2_	1.29–1.32, m	28.2, CH_2_	2.04, m	30.7^a^, CH_2_	1.29–1.32, m
	48	131.0, CH	5.35, m	30.8^b^, CH_2_	1.29–1.32, m	30.8, CH_2_	1.33, m	30.7^a^, CH_2_	1.29–1.32, m
	49	28.1, CH_2_	2.04, m	30.8^b^, CH_2_	1.29–1.32, m	30.1, CH_2_	1.32, m	30.3, CH_2_	1.29–1.32, m
	50	30.8, CH_2_	1.35, m	30.8^b^, CH_2_	1.29–1.32, m	32.9, CH_2_	1.30, m	33.1, CH_2_	1.29, m
	51	30.6, CH_2_	1.33, m	30.5, CH_2_	1.29–1.32, m	23.7, CH_2_	1.31, m	23.7, CH_2_	1.33, m
	52	32.9, CH_2_	1.30, m	33.1, CH_2_	1.27, m	14.4, CH_3_	0.90, t (6.9)	14.4, CH_3_	0.90, t (6.9)
	53	23.7, CH_2_	1.32, m	23.7, CH_2_	1.33, m				
	54	14.5, CH_3_	0.90, t (7.0)	14.4, CH_3_	0.90, t (7.0)				

a,b Unresolved carbon chemical shifts at δ_C_ 30.6(5), 30.7(4), 30.7(6), 30.7(7), and 30.8(0) for **2** and at δ_C_ 30.6(5) and 30.7(4) for **4**.

The molecular formula of delftibactin D (**2**) was determined to be C_54_H_94_N_14_O_19_ based on positive HRESIMS *m*/*z* 1243.6892 [M + H]^+^ (calculated 1243.6892, 0.0 ppm), presenting 16 degrees of unsaturation. Thorough examination of the 1D and 2D NMR data for compound **2** unveiled a close resemblance to the spectra of compound **1**, except for the absence of a double bond in the lipid tail for compound **2**. This was confirmed through the lack of olefinic signals evident in both the ^1^H NMR and ^13^C NMR spectra of compound **2**, as well as the presence of two additional methylene signals at δ_C_ 30.7 and 30.8 in the DEPT-Q NMR spectrum (Table 1 and Figures S13–S15). Moreover, tandem mass spectrometry demonstrated that each fragment containing the lipid tail in compound **2** exhibited an increase of 2 Da when compared to the equivalent fragment of compound **1** (Figures S11 and S12). Based on these findings, compound **2** was determined to be a tetradecanoic acid analogue of delftibactin A.

Delftibactin E (**3**) was obtained as a white powder and proved to have a molecular formula of C_52_H_88_N_14_O_19_ which was confirmed through positive HRESIMS *m*/*z* 1213.6414 [M + H]^+^ (calculated 1213.6423, −0.7 ppm), indicating 17 degrees of unsaturation. A thorough examination of the NMR data for compound **3** confirmed its resemblance to that of compound **1**. The sole disparity between compounds **3** and **1** lies in the length of the lipid tail, with compound **3** having a lipid tail two carbons shorter than that of compound **1**. This variation was corroborated through analysis of the HSQC cross peaks of the methylene groups in compound **3** (Figure S21). Moreover, the DEPT-Q NMR spectrum of compound **3** revealed 12 carbon signals for its lipid tail, indicating a variance of two carbon atoms when compared with the spectrum of compound **1**, which featured 14 carbon signals (Table 1 and Figure S20). Further support for this assignment is derived from the existence of lipid tail fragments in the MS/MS data for compound **3**, which are each 28 Da smaller than those in compound **1** (Figures S17 and S18). The double bond’s position was assigned through the observed HMBC correlations as with **1**. Cross peaks were detected from the methylene protons (H-42, δ_H_ 2.20) to the carbonyl (C-41, δ_C_ 175.3) and to two methylene carbons (C-43, δ_C_ 27.1 and C-44, δ_C_ 27.8) and from methylene protons (H-44, δ_H_ 2.08) to carbons C-43 (δ_C_ 27.1), C-42 (δ_C_ 36.8), and C-45 (δ_C_ 129.8) (Figures S22 and S30). Based on these findings, compound **3** was determined to be a 5-dodecenoic acid analog of delftibactin A.

The molecular formula of delftibactin F (**4**) was proved to be C_52_H_90_N_14_O_19_ through positive HRESIMS *m*/*z* 1215.6579 [M + H]^+^ (calculated 1215.6579, 0.0 ppm), implying 16 degrees of unsaturation. Thorough analysis of the ^1^H NMR, ^13^C NMR, and HSQC spectra of compound **4** (see Table 1 and Figures S27–S29) indicated high similarity to those of compound **2**. Nevertheless, a meticulous examination of the methylene groups in compound **4** revealed two fewer carbons in comparison to compound **2**. Additional supporting evidence was obtained from the observed lipid tail fragments in which the *m*/*z* values exhibited a reduction of 28 Da in compound **4** compared to those of compound **2** (Figures S25 and S26). Based on these findings, compound **4** was determined to be a dodecanoic acid analogue of delftibactin A.

We attempted to deduce the double-bond configuration of **1** and **3** through the *J* value of olefinic protons. Unfortunately, overlapping chemical shifts render it challenging to accurately measure the coupling constants. Nevertheless, the *cis* double-bond configuration was unequivocally ascertained from the δ_C_ values below 30 ppm (28.0 and 28.1 for **1** and 27.8 and 28.2 for **3**) of the allylic carbons adjoining the double bond (Table 1).^31^ Gunstone and colleagues demonstrated that the ^13^C NMR chemical shift can be used to distinguish between *cis* and *trans* configurations of alkenoic fatty acids.^31^ Following their rationale, we confidently identified the fatty acids in compounds **1** and **3** as (*Z*)-tetradec-7-enoic acid and (*Z*)-dodec-5-enoic acid, respectively.

### Identification of Delftibactin C–F BGC

A complete genome of *D. lacustris* was obtained by sequencing using a Nanopore MinION sequencer. Analysis of the *D. lacustris* genome using antiSMASH^27^ revealed a hybrid PKS-NRPS pathway on the chromosome which the tool assessed as 100% similar to the previously reported delftibactin A BGC. In this proposal we relied on the homology with the previously reported BGC that was investigated for its impact on the absolute configuration (e.g., to show the role and functionality of the standalone Asp β-hydroxylase DelD^32^). As we did not observe any products lacking the *N*-hydroxy or formyl groups, we hypothesize that the *N*-hydroxylase DelL and hydroxyornithine formyltransferase DelP act on the tethered peptide chain. However, further work is needed to confirm this. We also propose that the condensation domain of module six is a member of the dehydrating condensation domain (C_modAA_) class reported by Patteson and co-workers,^33^ here responsible for converting l-threonine into the dehydrobutyrine (Dhb) residue. However, further enzyme or bioinformatic analyses will be required to confirm this proposal.

### Absolute Configuration

**Figure 2:**
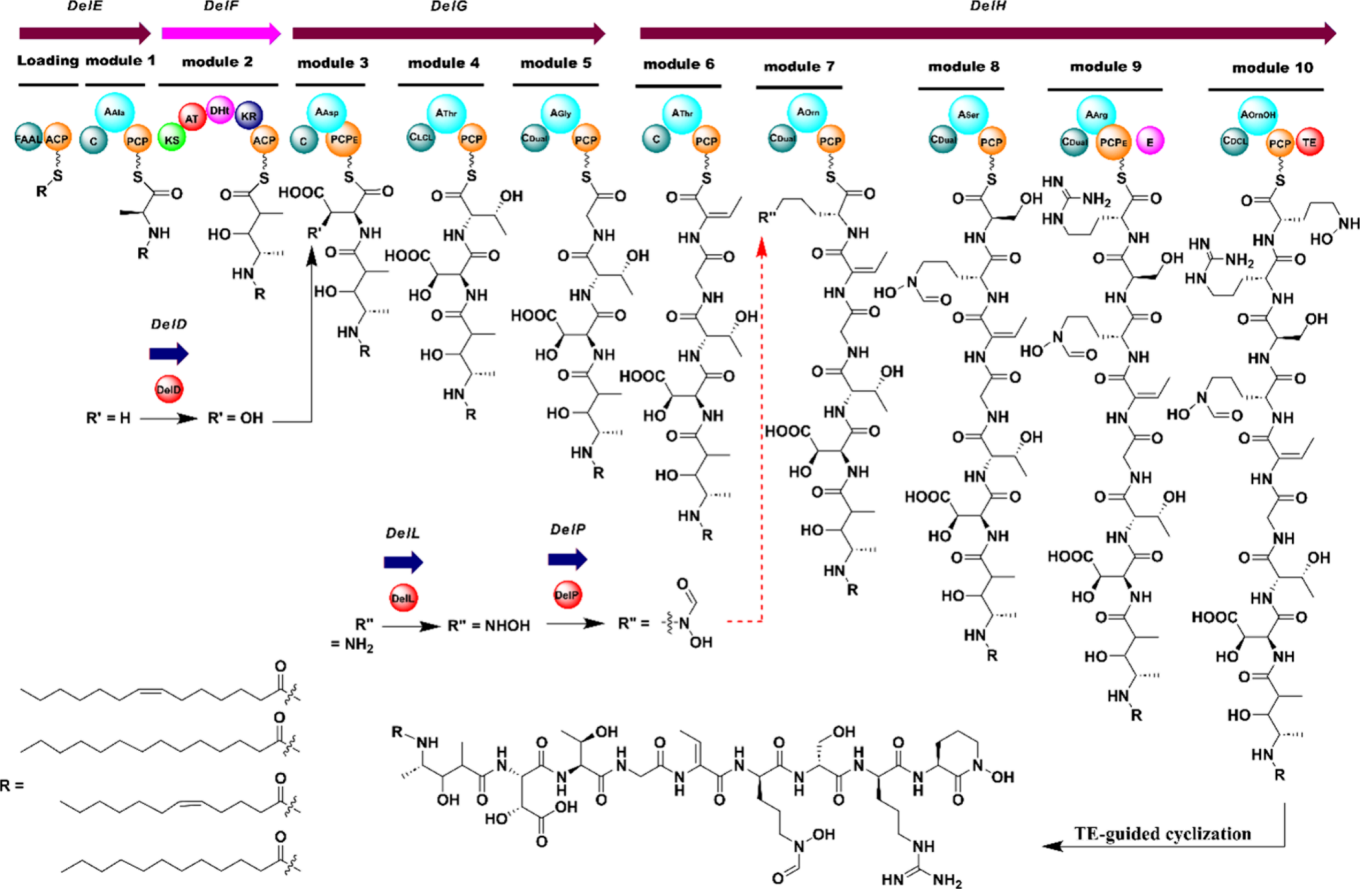
Proposed biosynthetic pathway of delftibactins C–F. Based on homology to the previously reported BGC and the absence of observed precursors, we propose the tailoring enzyme DelD (a standalone Asp β-hydroxylase) acts on the tethered chain, as previously reported.^32^ Based on the same reasoning, we also hypothesize that the *N-*hydroxylase DelL and the hydroxyornithine formyltransferase DelP also act on the tethered chain, but further work is needed to support this hypothesis. DelL and DelP could act after the completion of the linear scaffold. Note, arrows representing open reading frames are not drawn to scale. Domain notation: FAAL, fatty acid acyl ligase; ACP, acyl carrier protein; C, condensation; A, adenylation; PCP, peptidyl carrier protein (PCP_E_, specialized version of this domain followed by epimerase domains containing the “GGDSI” motif,^34^ also observed in the interaction with DelD^32^); KS, ketosynthase; AT, acyl transferase; DHt, dehydratase; KR, ketoreductase; C_Dual_, dual condensation; E, epimerase; and TE, thioesterase.

We performed Marfey’s analysis to determine the amino acid configurations in compound **1**.^35,36^ Compound **1** was subjected to hydrolysis with 55% HI and subsequently treated with l-FDAA, following the methodology described in a prior study.^32^ The resulting hydrolysate was compared with l-FDAA-derivatized amino acid standards with known configurations. This analysis revealed the configurations of the residues within **1** as follows: both an l-ornithine and a d-ornithine, d-arginine, d-serine, and l-threonine (Figures S31–S34). Regrettably, we were unable to detect the hydroxyaspartic acid isomer in our Marfey’s reaction product. For delftibactin A, the configuration of this amino acid (l-*erythro* isomer) was previously assigned by Reitz and co-workers based on a bioinformatic analysis of the responsible β-hydroxylase (DelD), which we hypothesized would be the same in our compounds.^32^ To validate this assignment, we examined our genome’s delftibactin BGC from the antiSMASH analysis. It has been reported that the β-hydroxylase (*delD*) gene in the delftibactin A BGC is responsible for the incorporation of the l-*erythro*-OH-Asp stereoisomer by hydroxylation of the loaded aspartic acid during the biosynthesis as a standalone enzyme.^32^ An equivalent gene was observed in our BGC by antiSMASH;^32^ alignment of this protein sequence with the original DelD showed only one amino acid difference (a >99% pairwise identity, see Table S1) in our *D. lacustris* genome. Based on this near-perfect homology, we propose that the configuration is conserved and l-*erythro* β-OH-Asp is also present in compound **1**. For the assignment of ornithine residues, our Marfey’s analysis indicated the presence of both configurations, confounding the assignment of configuration for these residues. In tackling this ambiguity, we undertook further genomic investigations. Initially, we explored the delftibactins’ BGC for epimerases. Regrettably, the only epimerase our annotation analysis recognized was on module 9, where its presence and the presence of a “GGDSI” motif in the peptidyl carrier protein (a PCP_E_ domain, the specialized version of this domain followed by epimerase domains^34^) were consistent with the epimerization of the serine residue to d-serine (SI Figure S35). No epimerase was identified that could explain the d-ornithine. To delve deeper into our genomic data, we performed a multiple sequence alignment of the amino acids between each pair of condensation domains in our detected delftibactin BGC and the previously reported dual condensation (C_Dual_) domains (Figure S36).^29,37,38^ We discovered four C_Dual_ domains in our BGC; the first, on module 5, was predicted to act on glycine, so it would not impact the absolute configuration. The second, on module 7, resolves the ambiguity of the ornithine moieties revealed in the Marfey’s analysis and indicated that the d-configuration of ornithine belongs to the internal moiety rather than the terminal one (the ornithine that becomes cyclized). The remaining C_Dual_ domains, on modules 8 and 9, affirmed the assignments of d-serine and d-arginine from our Marfey’s analysis, though we note that the serine could be acted on by either the C_Dual_ domain or the epimerase domain, discussed above, as both enzymes are predicted to convert the incorporated serine to d-serine. Based on this analysis and the homology with the previously reported BGC discussed earlier, we proposed a biosynthetic analysis for delftibactins C–F (Figure 2).

### Delftibactin C–Metal Interaction

To test the interaction of delftibactin C (**1**) with metals, aliquots of **1** were treated with various metal salts, including AuCl_3_, CuCl_2_, and FeCl_3_, and then analyzed by LCMS. A stable complex of the compound–iron adduct was formed in FeCl_3_-treated samples, which was confirmed by the complete loss of protonated delftibactin C at *m*/*z* 1241.6736 [M + H]^+^ and the formation of a new ion at *m*/*z* 1294.5855 [M – 2H + Fe(III)]^+^, which was consistent with the molecular formula of the ferric–compound complex (C_54_H_91_FeN_14_O_19_^+^, calcd 1294.5851, 0.3 ppm) (Figures S37 and S38). Complete conversion of the apo form to the iron adduct when mixing a 1:1 molar ratio of both compounds **1** and FeCl_3_ is consistent with the strong affinity of this molecule for iron predicted by its proposed role as a siderophore.^11^ The formation of an iron adduct with **1** was further verified by the observed maximum absorption at λ_max_ of 441 nm absent in apo-delftibactin C.^39,40^ Upon treatment of **1** with AuCl_3_, a series of unidentified ions emerged. We examined these new ions, for example, a new mass at *m*/*z* 1099.5980, but were unable to convincingly annotate these presumed oxidative degradation products of delftibactin C (Figures S39 and S40). Furthermore, treatment of compound **1** with soluble ionic gold led to the formation of a gold precipitate (observed upon centrifugation of the sample prior to LCMS screening), a phenomenon previously associated with delftibactin A (Figures S37 and S39). This shows that compound **1** possesses a gold biomineralization capability akin to that of delftibactin A, likely due to the presence of common structural features between the two compounds (Figure S39).^11^ Additionally, the interaction of compound **1** with CuCl_2_ revealed the loss of this compound (at *m*/*z* 1241.6736) and the emergence of a series of small unidentified ions suggestive of a potential for distinct chemistry with this metal (Figure S37). A screen using the Mass Query Language search tool (MassQL)^41^ revealed many copper adducts among these small ions (Table S2). However, we again encountered difficulty in annotating these ions, potentially due to oxidative–reductive chemistry interactions between the metal center and fragments of compound **1** that mask the original structure.

In summary, our thorough investigation into the metabolomics and genomics of *Delftia lacustris* DSM 21246 has resulted in the identification and structural characterization of four new amphiphilic metallophores, which we name delftibactins C–F (**1**–**4**). These compounds exhibit an integration of either saturated or unsaturated fatty acid components onto the delftibactin A peptide scaffold. The determination of their chemical structures was accomplished through NMR and MS/MS fragmentation techniques, while the absolute configuration of the peptide backbone was assigned using Marfey’s and bioinformatic analyses. Additionally, compound **1** was tested for its stability in the presence of different metal salts, including iron, gold, and copper. These evaluations revealed the formation of an iron adduct of **1**. In the case of gold, the color change along with gold precipitate formation indicated that this lipid-bearing analog of delftibactin A retains the previously reported gold-biomineralizing ability.^11^ However, with copper, a series of unidentified small Cu-binding product ions were detected, hinting at different chemistry. In sum, compound **1** forms distinct products when reacted with different metal salts, which raises our interest in this selectivity and its potential use in the bioremediation of heavy metals. Further investigation will be required to determine the nature of these compounds’ chemistry with copper and measure the capacity of these compounds for remediation of heavy metals in polluted environments.

## Experimental Section

### General Experimental Procedures

UV absorption maxima were measured using a UV-1600PC spectrophotometer (VWR). IR spectra were recorded on an Agilent Technologies Cary 630 FTIR. 1D- and 2D-NMR experiments were carried out on a Bruker Avance III-500 MHz spectrometer using CD_3_OD as solvent with TMS as internal standard (Cambridge Isotope Laboratories). HRESIMS and MS/MS spectra were collected using an Agilent 6530C Q-TOF LC/MS system with an Agilent Jet Stream source and an Agilent 1260 Infinity II HPLC pump stack. RP-SPE C18 columns (100 mg, 1000 mg, and 5 g, Thermo Scientific) were used for the purification steps before HPLC. For the HPLC purification, an Agilent 1260 Infinity II HPLC with multiple wavelength detector and fraction collector was used. Analytical grade solvents (Fisher and VMR) were used for the isolation and purification procedures. LCMS grade solvents (Honeywell CHROMOSOLV LCMS water, or redistilled MilliQ purified water, and Supelco LiChrosolv LCMS acetonitrile) buffered with formic acid (puriss Sigma-Aldrich) were used for LCMS runs. New Brunswick Innova 4430 shaker incubators were used for bacterial cultivation. Media was prepared using Millipore Sigma products, except for the pyruvic acid, which came from BeanTown Chemical (BTC), in Milli-Q purified water. Frozen stocks of the strain were stored in a VWR brand ultralow temperature freezer set to −70 °C. For the Marfey’s analysis, the *N*-(5-fluoro-2,4-dinitrophenyl)-l-alaninamide came from TCI America, while the high-quality amino acid standards were from the following suppliers: l-ornithine hydrochloride (Acros), d-ornithine hydrochloride (Alfa Aesar), d-allothreonine (TCI), dl-allothreonine (TCI), d-threonine (BTC), l-threonine (ICN), dl-arginine hydrochloride (TCI), l-arginine (Sigma-Aldrich), d-serine (BTC), and l-serine (ICN).

### Bacterial Fermentation

The bacterial strain *D. lacustris* DSM 21246 was purchased from the German Collection of Microorganisms and Cell Cultures (DSMZ) as a lyophilized stock. After rehydration of the stock with liquid lysogeny broth and plating on NRRL-1 medium, a single colony was picked from this plate into a 5 mL starter culture of our lab’s DMS. Briefly, this media was prepared with 0.30 g/L KH_2_PO_4_, 0.30 g/L MgSO_4_, 1 g/L citric acid, 2.00 g/L l-glutamine, and 2.00 g/L 3-(*N*-morpholino)propanesulfonic acid (MOPS) in deionized water, adjusted to a pH of 7.5 with 1.0 M NaOH(aq).^24^ The starter culture was grown to turbidity, and then a 1:1 culture to 50% glycerol stock was prepared and frozen at −70 °C for long-term storage of the strain. To restart the culture, the *D. lacustris* frozen stock was first restreaked on solid agar plates of DMS (with 15 g/L agar). Then, after sufficient growth, a single colony was inoculated into liquid culture at a 5 mL scale; this starter culture would be used to inoculate either the biological replicates for the iron supplementation experiment or larger scale media preparations for the isolation work.

### Metabolomic Profiling

#### Iron Limitation Experiment

In duplicate, 5.0 μL of the starter culture was inoculated into 5.0 mL of fresh DMS supplemented with either 50 μL of a filter-sterilized solution containing 0.12 g of citric acid monohydrate in 200 mL of deionized water (for the iron-depleted condition) or 50 μL of a filter-sterilized solution containing 0.12 g of citric acid monohydrate and 0.12 g of ferric ammonium citrate in 200 mL of deionized water (for the iron-replete condition). The addition of the ferric ammonium citrate solution led to a final iron concentration of 23 nM in the iron-replete culture broth. After incubating these cultures for 3 days, they were centrifuged (for 6 min at 21 000 rcf and 13 °C) to separate the bacterial cells, and the resulting supernatants were passed through 100 mg C18 RP-SPE cartridges. The metallophores were found in the 50% aqueous MeCN fraction following elution with 1000 μL each of Milli-Q H_2_O, 50% aqueous MeCN, and MeCN as eluting agents. Subsequently, the 50% MeCN fractions were subjected to LCMS analysis to compare the two conditions.

#### Liquid Chromatography and Mass Spectrometry Method

The LCMS pump stack was equipped with a core–shell Kinetex, 2.6 μm 50 × 2.1 mm 100 Å EVO C_18_ column (Phenomenex). The LC gradient pump method used 0.1% formic acid-acidified H_2_O (redistilled) as solvent A and 0.1% formic acid-acidified MeCN as solvent B with the following program: a starting elution of 10% of B for 3 min, linear gradient to 25% B over 5 min, then a linear gradient to 99% B over 7.5 min, then held for 3 min at 99% B, before returning to the starting elution over 2 min, and a reequilibration for 2.5 min. This program was run with a flow rate of 450 μL/min and a 10 μL injection volume.

#### GNPS Analysis of the MS/MS Data

Using the Global Natural Product Social Molecular Networking web platform (GNPS), a molecular network was constructed.^25^ The network incorporated duplicate runs from the high/low-iron experiment, and no injection blanks were run before each of these experiments. The network was generated with a small data preset as the networking parameter using the following settings: A minimum matrix fragment ion setting of 6, a minimum cluster size setting of 2, and a cosine score setting of 0.55. To ensure the accuracy of the assignments, manual annotation of all fragment spectra was performed. The details of this validation process can be found in SI Figures S3, S4, S11, S12, S17, S18, S25, and S26 and Table 1.

### Isolation of Delftibactins C–F

For metallophore isolation, 1 mL of the starter culture was added to 1 L of liquid DMS in a 2.8 L baffled Erlenmeyer flask in four separate batches. The bacterium was allowed to grow shaking at 180 rpm on a rotary shaker at 30 °C for about 48 h. The cultures (4 × 1 L) were harvested by shaking them with HP-20 resin (20 g/L at 180 rpm for 2 h using an orbital shaker). This suspension was filtered through filter paper to remove the culture supernatant and cells; then the remaining resin was washed with 0.5 L of Milli-Q water. Finally, the adsorbed metabolites were eluted using 4 × 100 mL of methanol. This methanol extract was concentrated by rotary evaporation, and the presence of the metallophores was confirmed using LCMS analysis. The crude extract was subsequently fractionated by RP-SPE with a 5 g C_18_ column using 20 mL each of H_2_O, 50% aqueous MeCN, and MeCN. The 50% MeCN fraction was purified via RP-HPLC using a C_18_ semipreparative (Phenomenex Luna, 250 × 10 mm, 5 μm) column. The following solvents were used: H_2_O acidified with 0.1% formic acid (solvent A) and MeCN acidified with 0.1% formic acid (solvent B). A linear gradient from 45% to 75% of solvent B over 15 min was used to afford four fractions. These fractions were concentrated to reduced volume by rotary evaporation and then reduced to dryness on a freeze-dryer. The first fraction was further purified on RP-HPLC to isolate compound **3** using a gradient from 35% to 55% of solvent B over 15 min. The method was slightly modified, using a gradient from 45% to 62% of solvent over 15 min, to facilitate the isolation of compound **4** from the second fraction. Compounds **1** and **2** were isolated from the third and fourth fractions, respectively, using a gradient method from 45% to 75% of solvent B over 25 min.

#### Delftibactin C (**1**)

Yellowish white powder; [α]^22.6^_D_ −20 (*c* 0.1, MeOH); IR (ν_max_, MeOH) 3278, 2931, 1638, 1526, 1349, 1095 cm^–1^; ^1^H NMR (500 MHz, CD_3_OD) and ^13^C NMR (125 MHz, CD_3_OD) are shown in Table 1; HRESIMS (positive mode) *m*/*z* 1241.6730 [M + H]^+^ (calcd for C_54_H_93_N_14_O_19_^+^, 1241.6736, 3.9 ppm).

#### Delftibactin D (**2**)

Yellowish white powder; [α]^22.4^_D_ −40 (*c* 0.1, MeOH); IR (ν_max_, MeOH) 2985, 2920, 1638, 1533, 1354, 1094 cm^–1^; ^1^H NMR (500 MHz, CD_3_OD) and ^13^C NMR (125 MHz, CD_3_OD) are shown in Table 1; HRESIMS (positive mode) *m*/*z* 1243.6892 [M + H]^+^ (calcd for C_54_H_95_N_14_O_19_^+^, 1243.6892, 4.1 ppm).

#### Delftibactin E (**3**)

Yellowish white powder; [α]^22.4^_D_ +10 (*c* 0.1, MeOH); IR (ν_max_, MeOH) 3311, 2942, 1638, 1522, 1353, 1099 cm^–1^; ^1^H NMR (500 MHz, CD_3_OD) and ^13^C NMR (125 MHz, CD_3_OD) are shown in Table 1; HRESIMS (positive mode) *m*/*z* 1213.6414 [M + H]^+^ (calcd for C_52_H_89_N_14_O_19_^+^, 1213.6423, 3.6 ppm).

#### Delftibactin F (**4**)

Yellowish white powder; [α]^22.4^_D_ −30 (*c* 0.1, MeOH); IR (ν_max_, MeOH) 3272, 2924, 1647, 1526, 1351, 1097 cm^–1^; ^1^H NMR (500 MHz, CD_3_OD) and ^13^C NMR (125 MHz, CD_3_OD) are shown in Table 1; HRESIMS (positive mode) *m*/*z* 1215.6579 [M + H]^+^ (calcd for C_52_H_91_N_14_O_19_^+^, 1215.6579, 0.0 ppm).

### Marfey’s Analysis

Approximately 1.5 mg of **1** was dissolved in 200 μL of Milli-Q water, and 200 μL of 55% HI(aq) added. The acidified solution was transferred to a one-dram vial, and the cap was sealed tightly with Parafilm. The sealed vessel was heated for 22 h at 100 °C in a sand bath, and then the crude hydrolysate was transferred to a fresh vial and evaporated by nitrogen gas flow. The dried material was repeatedly redissolved in 700 μL of Milli-Q water, dried again (three times total) to remove any residual acid, and then brought to a final volume of 100 μL in Milli-Q water. The hydrolysate was reacted with l-DAA (Marfey’s reagent) following literature conditions^32^ and subjected to HPLC with the following program: 90% solvent A and 10% of solvent B for 6 min; linear gradient to 25% B over 9 min; linear gradient to 55% B over 5 min; gradient to 99% B for 2 min; then for 4 min at 99% B; return to the starting elution over 2 min and a reequilibration for 6 min with a flow rate of 450 μL/min and 10 μL injection volume.

### Genome Mining

#### Genomic DNA Isolation

Genomic DNA, both high and low molecular weight (HMW and LMW), was isolated from cultures of *D. lacustris* bacterium in 5 mL of DMS medium grown for 2 days at 28 ^ο^C, with 5 mL of the culture used for each separate DNA extraction procedure. Following the incubation period, the bacterial cells were collected by centrifuging the entire culture broth at 21 000 rcf and 13 °C for 5 min. After removal of the culture supernatants, the bacterial cells were subjected to DNA isolation. The NucleoBond HMW DNA kit (Macherey-Nagel) was employed for HMW DNA, following the manufacturer’s protocol with a minor modification. Initially, the bacterial pellet underwent lysis using the bacterial cell lysis protocol as utilized in the OMEGA Bio-Tek E.Z.N.A. bacterial DNA kit. For this step, TE buffer (100 μL) and lysozyme (10 μL) were added to the bacterial cell pellet, and this mixture was allowed to incubate for 10 min. Following this incubation period, an addition of TL buffer (100 μL) and proteinase K (20 μL) was made, followed by an hour-long incubation at 65 °C. Next, 5 μL of RNase was introduced into the tube and kept at room temperature for 5 min. Then subsequent to the lysis stage, the HMW DNA from *D. lacustris* was isolated in accordance with the instructions detailed in the protocol of the NucleoBond HMW DNA kit. LMW DNA was also extracted from a separate bacterial cell pellet following the manufacturer’s protocol of the OMEGA Bio-Tek E.Z.N.A. bacterial DNA kit.

#### Library Preparation and Whole Genome Sequencing

For nanopore DNA sequencing, the DNA extracts were processed separately to create two DNA libraries using the ligation sequencing kit (SQK-LSK109, Oxford Nanopore Technologies Inc.) following the manufacturer’s protocol. The prepared HMW DNA library was loaded into a MinION flow cell (FLO-MIN111 R10.3 version) and sequenced on a MinION device (Oxford Nanopore Technologies Inc.). After utilization, the flow cell underwent cleaning with the flow cell wash kit (Oxford Nanopore Technologies Inc.) and was subsequently stored at a temperature of 4 °C. Following a wash with the flow cell wash kit, the same flow cell was reused for sequencing the LMW DNA library, which was also prepared with the LSK 109 kit.

#### Bioinformatic Workflow

Following the completion of the nanopore sequencing runs, the generated. fast5 data for both HMW and LMW DNA sequencing were transferred to the computational resources within the GlyCORE Computational Chemistry and Bioinformatics Core basecalling processing and genome assembly. The initial step involved basecalling the .fast5 nanopore reads utilizing the Guppy basecaller (version 6.4.2), employing the superaccuracy configuration file tailored to our specific kit and flow cell. Once the reads were successfully basecalled and converted to fastq files, a pycoQC report was generated to evaluate the quality of the reads. To eliminate adapter sequences from the initial basecalled reads, the Porechop tool was employed.^42^ Subsequently, these adapter-free sequences underwent processing using the FiltLong tool.^43^ For the FiltLong configuration, we established the minimum length threshold at 500 bp, and the quality score was set to exclude at least 1% of reads based on their quality (but this step occurred after short read removal, so in effect the reads were only trimmed for minimum length). Upon the completion of the FiltLong process, an initial draft assembly of one circular chromosome and one circular plasmid was generated utilizing Flye (version 2.9).^44^ Medaka was used to improve the draft Flye assembly.^45^ For the Medaka run the inputs were the draft Flye assembly and a different reads file produced by FiltLong trimming of the raw base, called reads below 1000 bp, and a removal of the 10% by quality score exclusion. The Medaka-polished Flye-assembled genome of *D. lacustris* DSM 21246 was deposited in NCBI GenBank, with accession numbers CP141274 and CP141273 assigned to our assembly of the 7 Mb chromosome and 302 kb plasmid, respectively. This genome was mined for potential biosynthetic gene clusters using antiSMASH bacterial version 7.0.1.^27^

#### Stability of Delftibactin C with Metals

A stock 1 mM solution of compound **1** was made in Milli-Q water. Similarly, stocks of 1 mM solutions of the separate metal salts (AuCl_3_, CuCl_2_, or FeCl_3_) were prepared. Separately, in duplicate, equal volumes (100 μL) of compound **1** stock solution and each metal solution were mixed. These mixtures were kept at room temperature for 1 h to allow the interaction between the compound and the metals. After the incubation period, the samples were centrifuged to remove any particulates. Samples then were fractionated on RP-C18 SPE cartridges (100 mg) using H_2_O, 50% MeCN in H_2_O, and MeCN as eluents. Fifty percent of the MeCN fractions were then analyzed by LCMS.

## Data Availability

The genome sequence is available under the following BioProject number PRJNA1054122 with the accession numbers CP141274 and CP141273 assigned to our assembly of the chromosome and plasmid, respectively. The LCMS data for the crude extract of *D. lacustris* used for delftibactin C–F isolation, the iron limitation experiment, and the stability of compound **1** with iron, gold, and copper have been uploaded to the GNPS-MassIVE archive with accession ID: MSV000093629. Raw NMR data have been submitted to the Natural Products Magnetic Resonance Database (NP-MRD) (https://np-mrd.org) and are available with the following IDs for compounds **1**–**4**, respectively: NP0332726, NP0332727, NP0332728, and NP0332729.

## Supporting Information Available

MS instrument details, MS and NMR data, and genomic analysis (PDF)
