# Supplementary material for: LCMS-Metabolomic Profiling and Genome Mining of Delftia lacustris DSM 21246 Revealed Lipophilic Delftibactin Metallophores
Source: J Nat Prod. 2024 May 13;87(5):1384–93. doi: 10.1021/acs.jnatprod.4c00049 (PMC11472818; doi:10.1021/acs.jnatprod.4c00049)
Supplement: Supplementary file 1 — np4c00049_si_001.pdf [file np4c00049_si_001.pdf]

SUPPLEMENTARY INFORMATION

LCMS-Metabolomic Profiling and Genome Mining of *Delftia lacustris* DSM 21246 Revealed Lipophilic Delftibactin Metallophores

Mohammed M. A. Ahmed[1,2] and Paul D. Boudreau[1*]

[1]Boudreau Lab, Department of BioMolecular Science, School of Pharmacy, University of Mississippi, University, MS, USA

[2]Department of Pharmacognosy, Al-Azhar University, Cairo, Egypt

* Corresponding Author:

Email boudreau@olemiss.edu (Paul D. Boudreau)

Table of Contents

**LCMS Method Details**	…………………………………………………………………………………………	3
**Figure S1.**	LCMS-based Metabolomic Profile of *Delftia lacustris* DSM 21246………………………………………	4
**Figures S2-9.**	IR, HRMS spectra, fragment structures, and NMR spectra for compound **1**………………………………	5
**Figures S10-15.**	IR, HRMS spectra, fragment structures, and NMR spectra for compound **2**………………………………	12
**Figures S16-23.**	IR, HRMS spectra, fragment structures, and NMR spectra for compound **3**………………………………	17
**Figures S24-29.**	IR, HRMS spectra, fragment structures, and NMR spectra for compound **4**………………………………	24
**Table S1.**	Table of ^{1}H and ^{13}C NMR Chemical Shifts of **1 – 4**……………………………………………	29
**Figure S30.**	Key HMBC and TOCSY correlations of **1** and **3**………………………………………………	31
**Figures S31-34.**	Marfey's analysis of compound **1**…………………………………………………………	32
**Table S1, Figure S35, S36.**	Genome and delftibactin BGC sequence analysis ………………………………………………	34
**Figure S37.**	Interaction of delftibactin C (**1**) with metals (iron, gold, and copper) ………………………………	37
**Figure S38.**	Formation of an iron adduct of delftibactin C (**1**) with iron …………………………………………	38
**Figure S39, S40.**	Formation of a gold precipitate and an oxidative degradation product of delftibactin C (**1**) with gold…	39
**Table S2.**	MASSQL search terms for copper adducts………………………………………………………	41

LCMS Method Details:

Centroid data was collected with an Auto MS/MS method collected in static positive ion polarity with absolute storage thresholds of 200 and 5 counts for the MS and MS/MS scans, respectively. The source settings were: drying and sheath gas temperatures of 300 and 325 °C, respectively; drying and sheath gas flow rates of 10 and 3 L/min, respectively; a nebulizer pressure of 50 psi; and capillary and nozzle voltages of 4000 and 0 V, respectively. The ion optics were set with the fragmentor at 200 V, the skimmer at 65 V, and the octopole 1 RF Vpp at 750 V. In the experiment segment from 3.0 to 17.5 minutes the Auto MS/MS settings were: An MS scan over the mass range of 100-1600 *m/z* with an acquisition rate of 5 spectra/s with an MS/MS scan over the mass range of 100-1305 *m/z* with an acquisition rate of 3 spectra/s. The MS/MS collision energy was set at a gradient with the following formula: 3(*m/z*)/100 + 15. For the MS/MS acquisition, a maximum of 6 precursor per cycle was used, with static exclusion below 250 and above 1300 *m/z*, or below an absolute threshold of 10000 counts and active exclusion after 3 spectra within a 0.2-minute range. Reference mass signals and some common contaminants were also added to an exclusion list for the entirety of the run (922.0098, 531.40777, 553.38972, 1083.791 *m/z* at a range of 100 ppm).

Figure S1: LCMS-based Metabolomic Profile of *Delftia lacustris* DSM 21246.

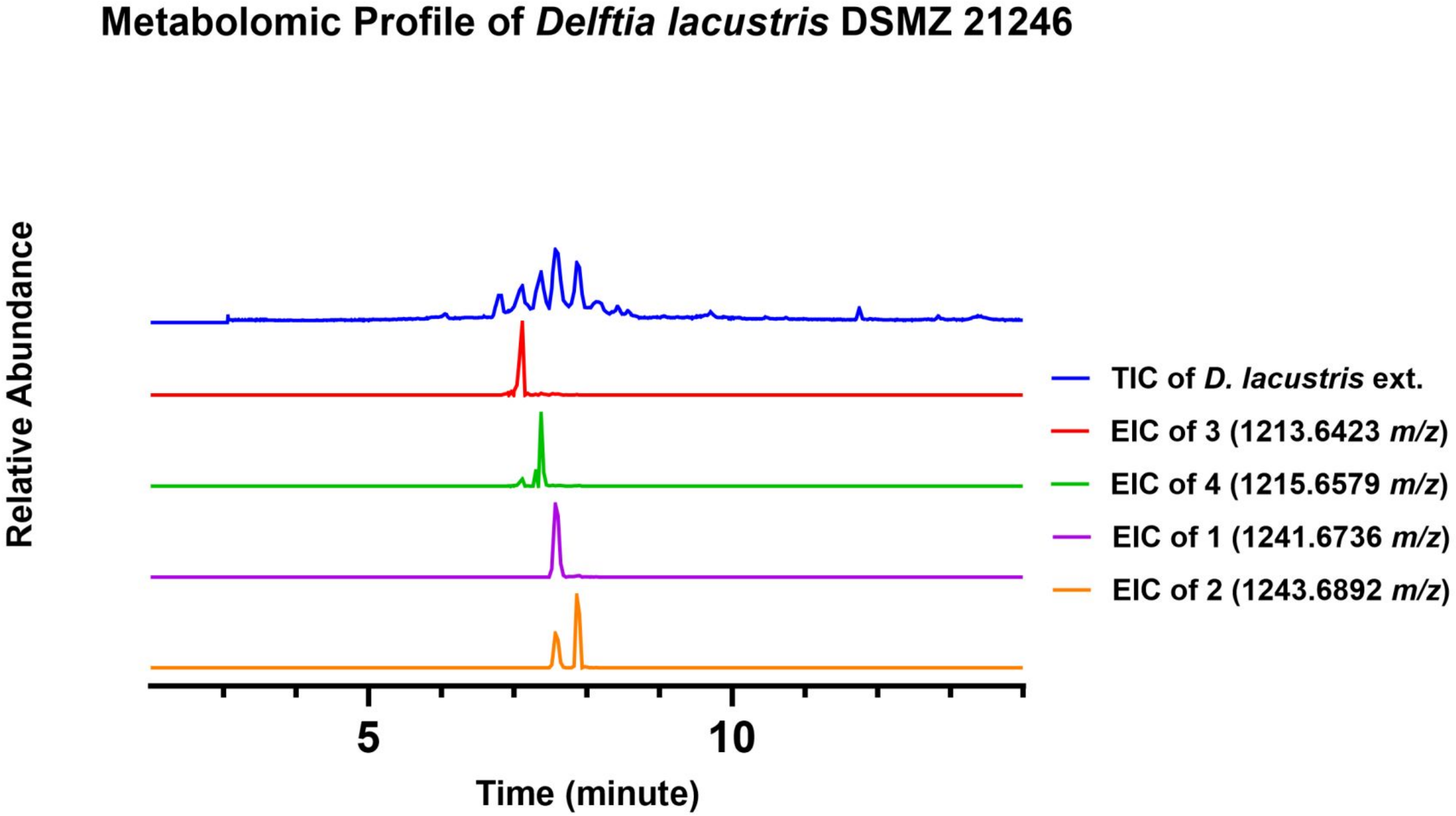

This figure shows the total ion chromatogram (TIC) of the crude extract of *D. lacustris* 21246 cultured on our Defined Medium for Siderophore (DMS) (**Blue**), and the extracted ion chromatogram (EIC) for compounds **1** – **4** (**Red, Green, Purple, Orange,** respectively).

Figure S2: IR Spectrum of 1

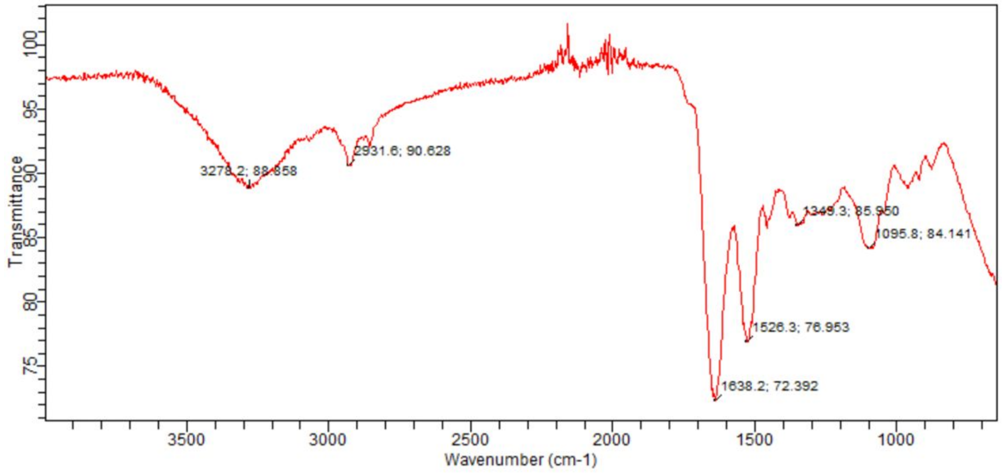

Figure S3: HRMS^2 Fragment Spectrum of 1

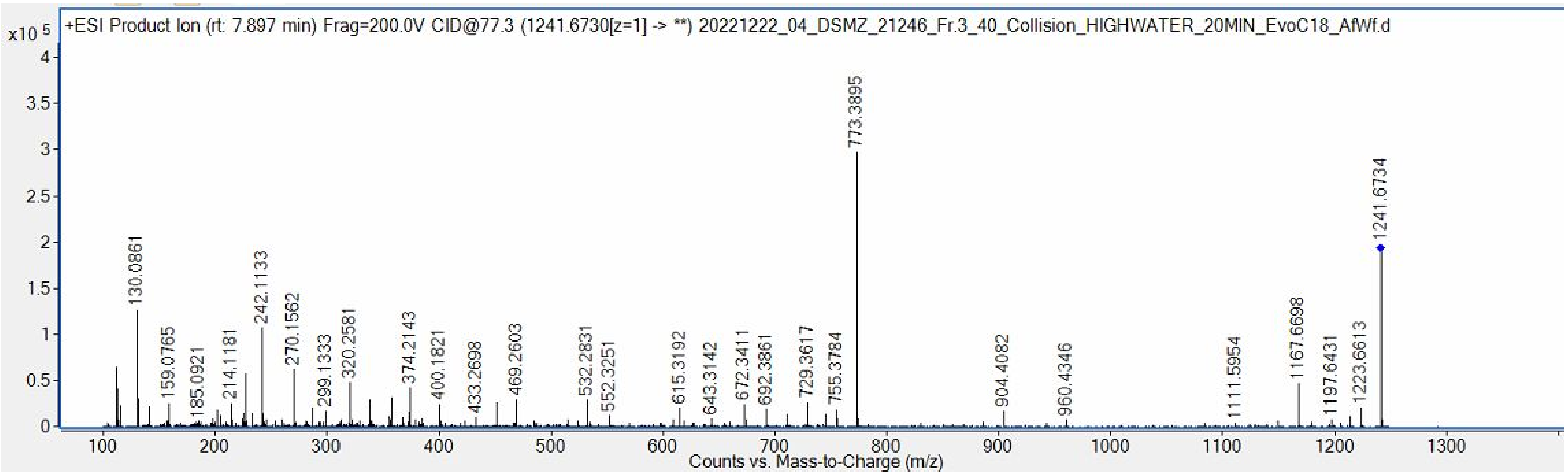

Figure S4: Fragment Structures of 1

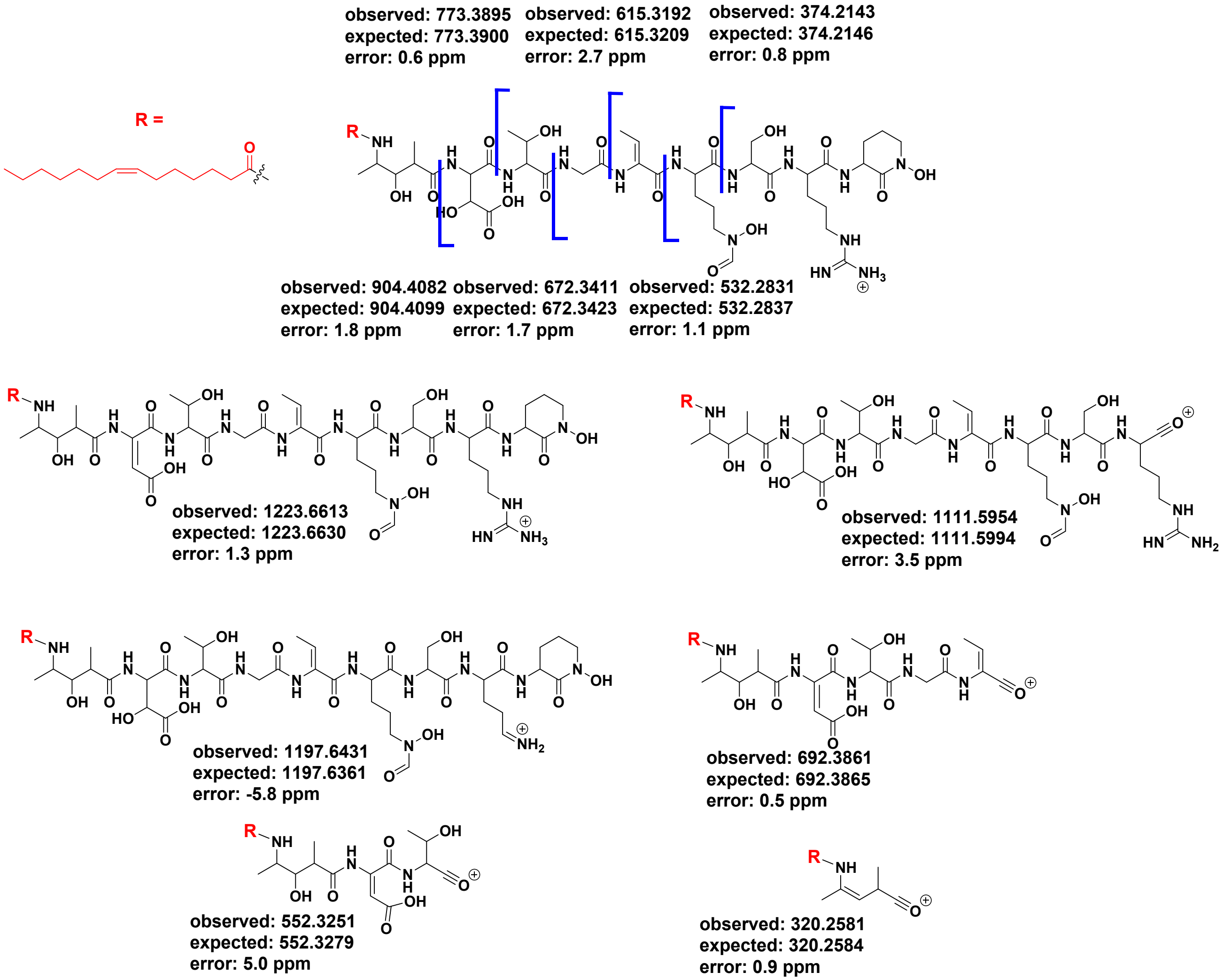

Figure 5: ^{1}H-NMR (500 MHz, CD_3OD) Spectrum of 1

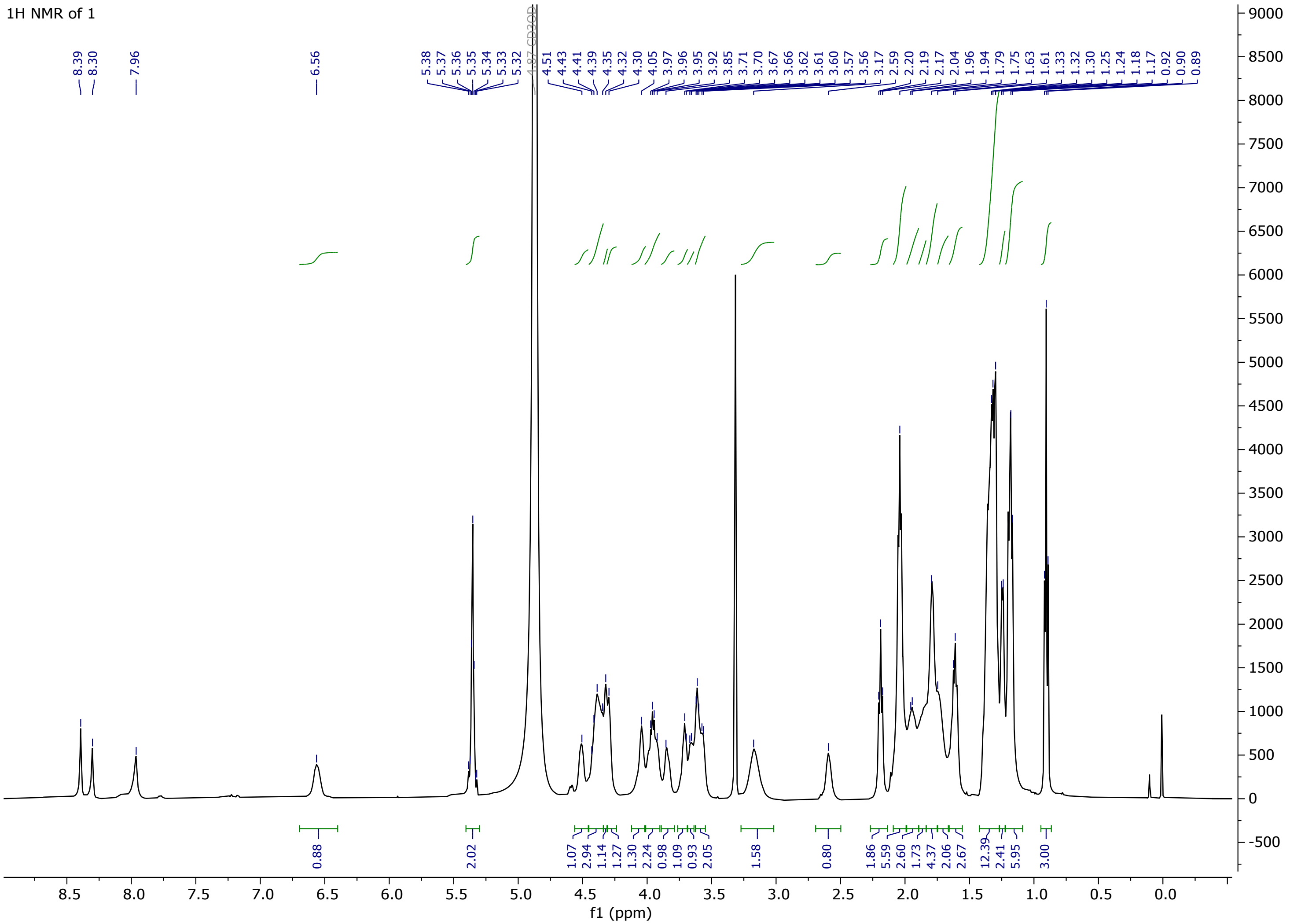

Figure 6: Dept-Q ^{13}C-NMR (125 MHz, CD_3OD) Spectrum of 1

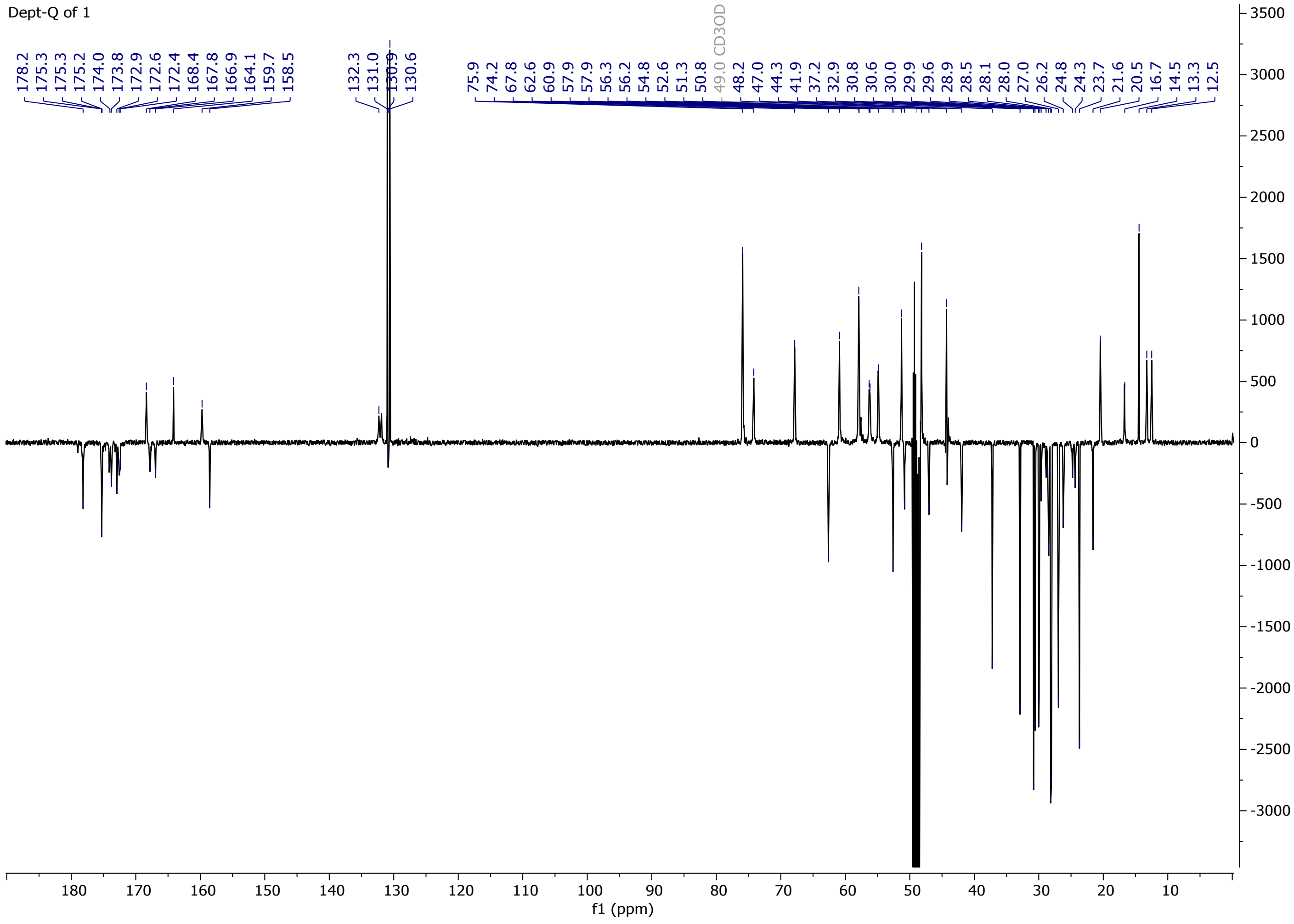

Figure 7: HSQC Spectrum of 1

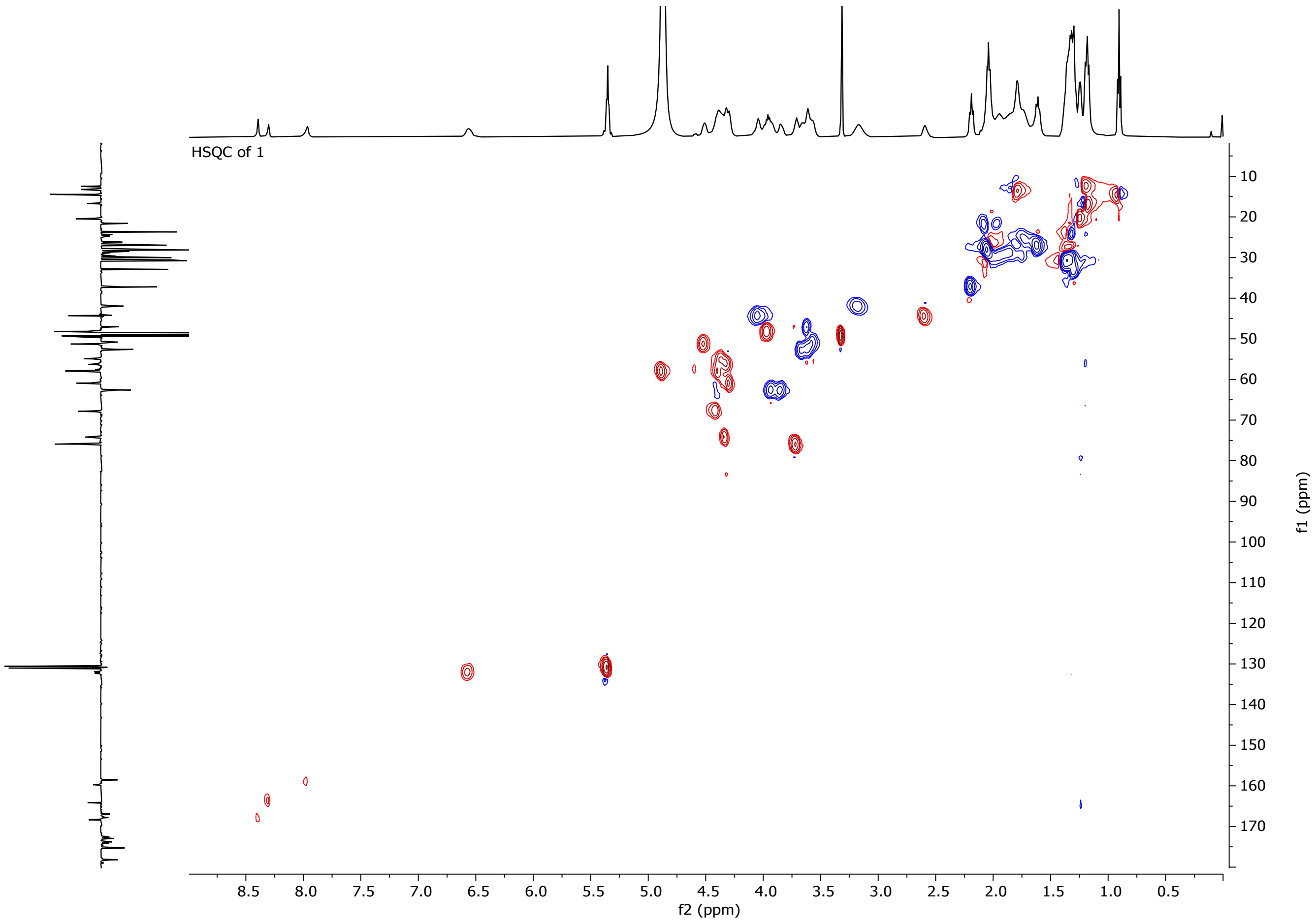

Figure 8: HMBC Spectrum of 1

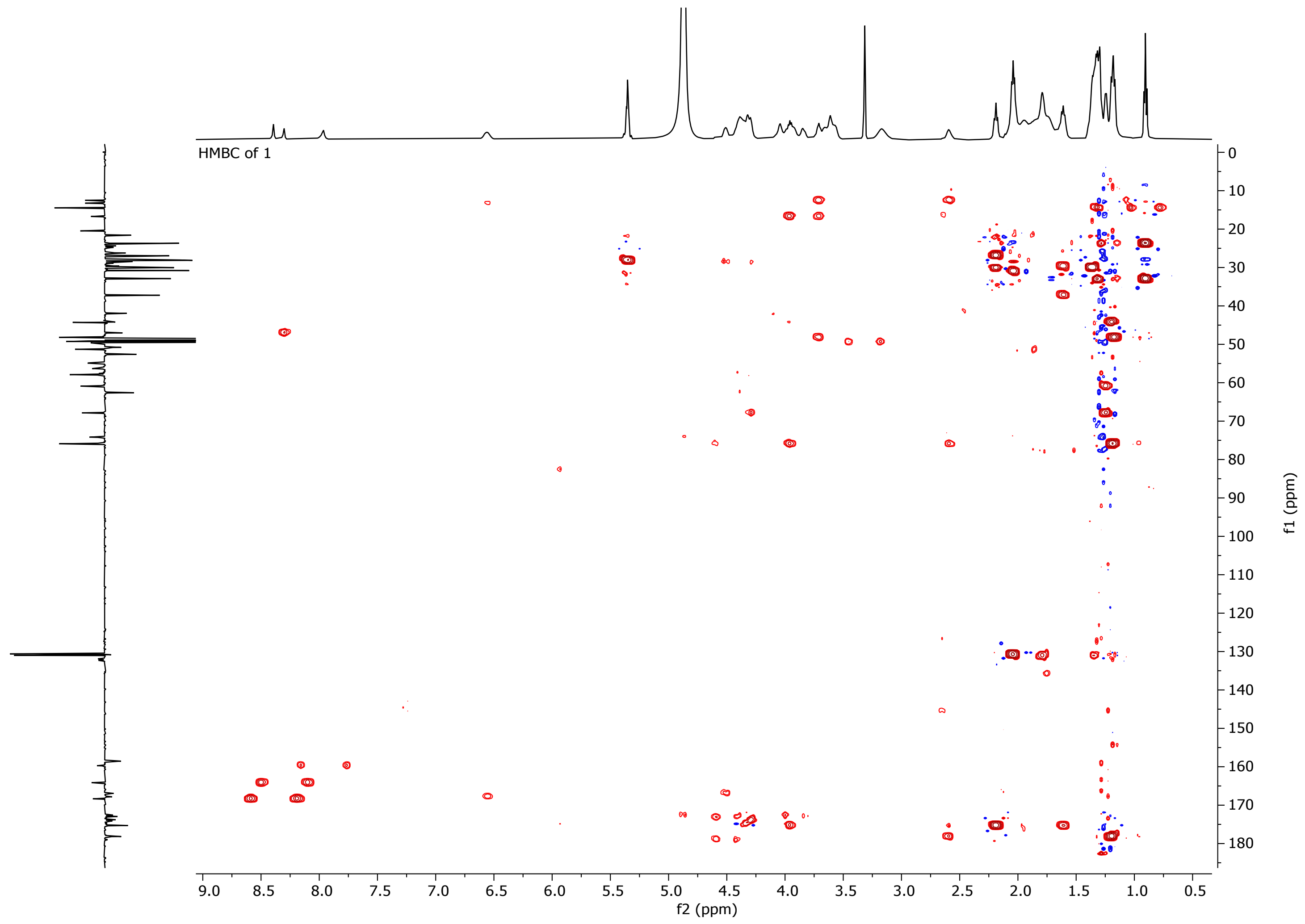

Figure 9: ^{1}H-^{1}H TOCSY Spectrum of 1

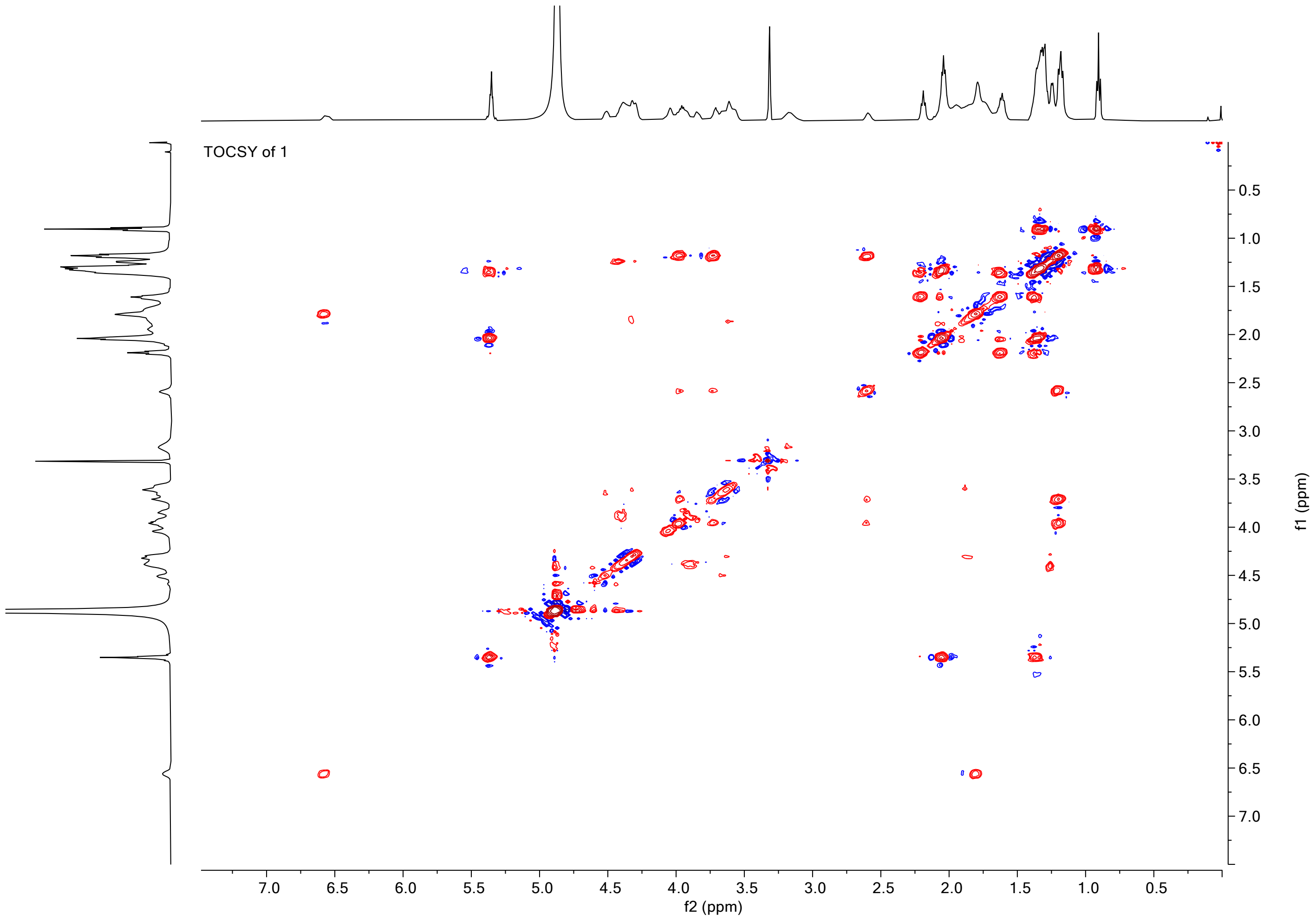

Figure 10: IR Spectrum of 2

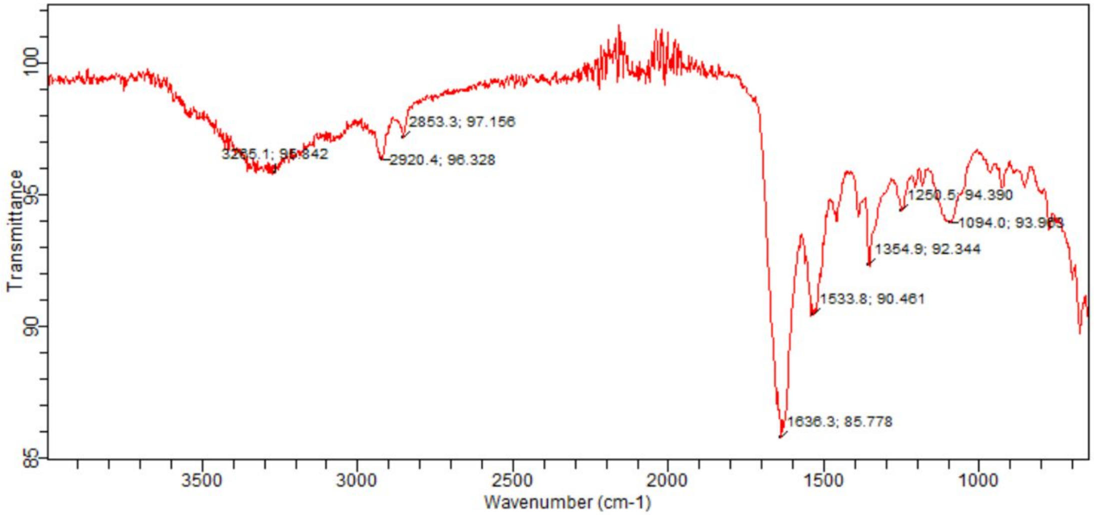

Figure 11: HRMS^2 Fragment Spectrum of 2

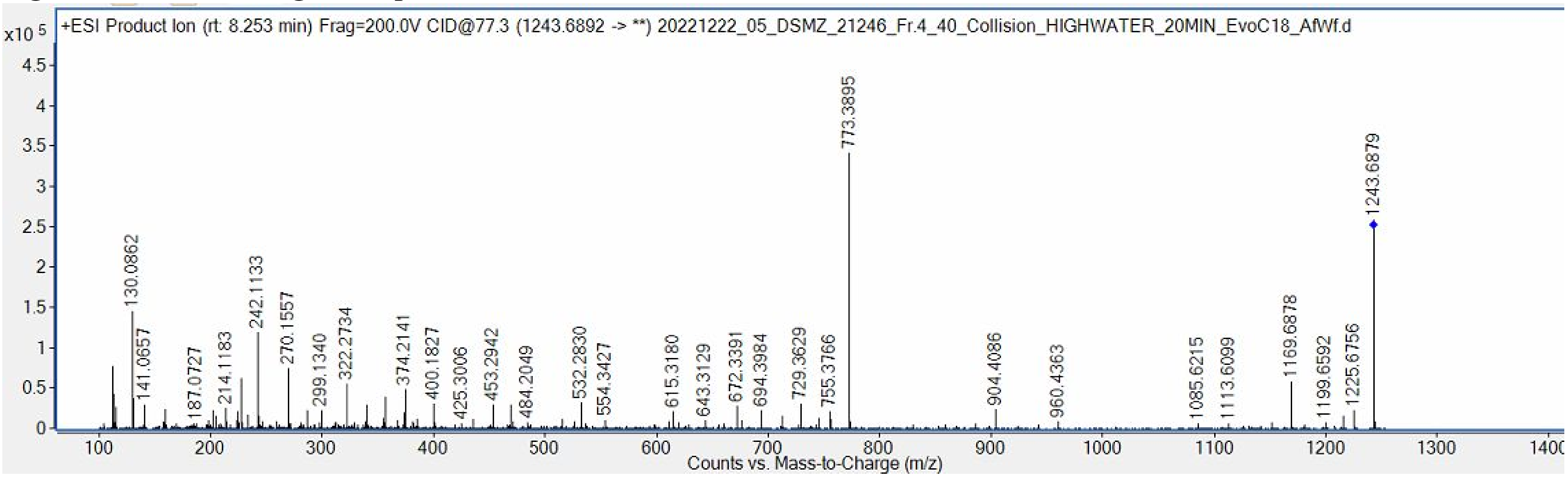

Figure 12: Fragment Structures of 2

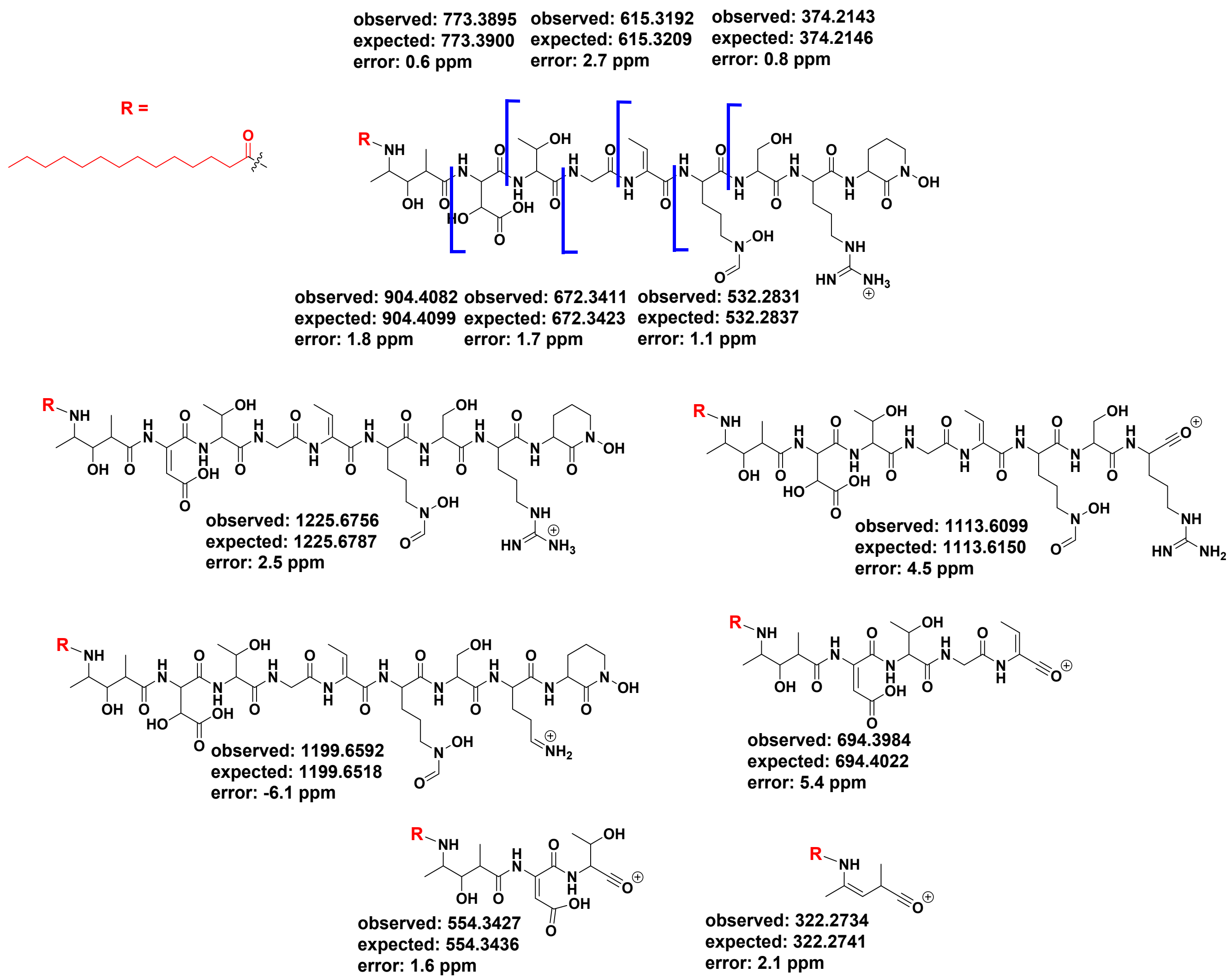

Figure 13: ^{1}H-NMR (500 MHz, CD_3OD) Spectrum of 2

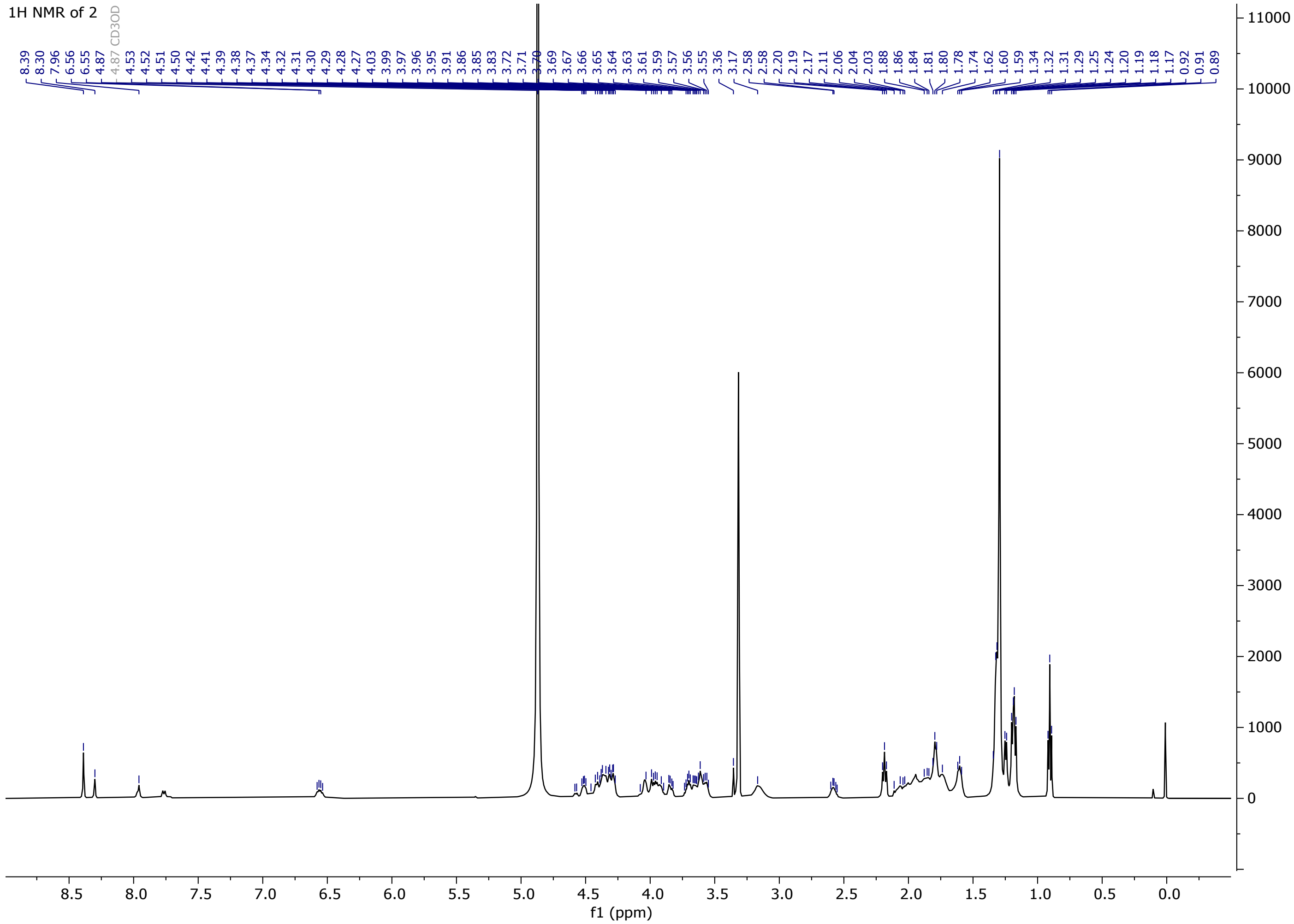

Figure 14: Dept-Q ^{13}C-NMR (125 MHz, CD_3OD) Spectrum of 2

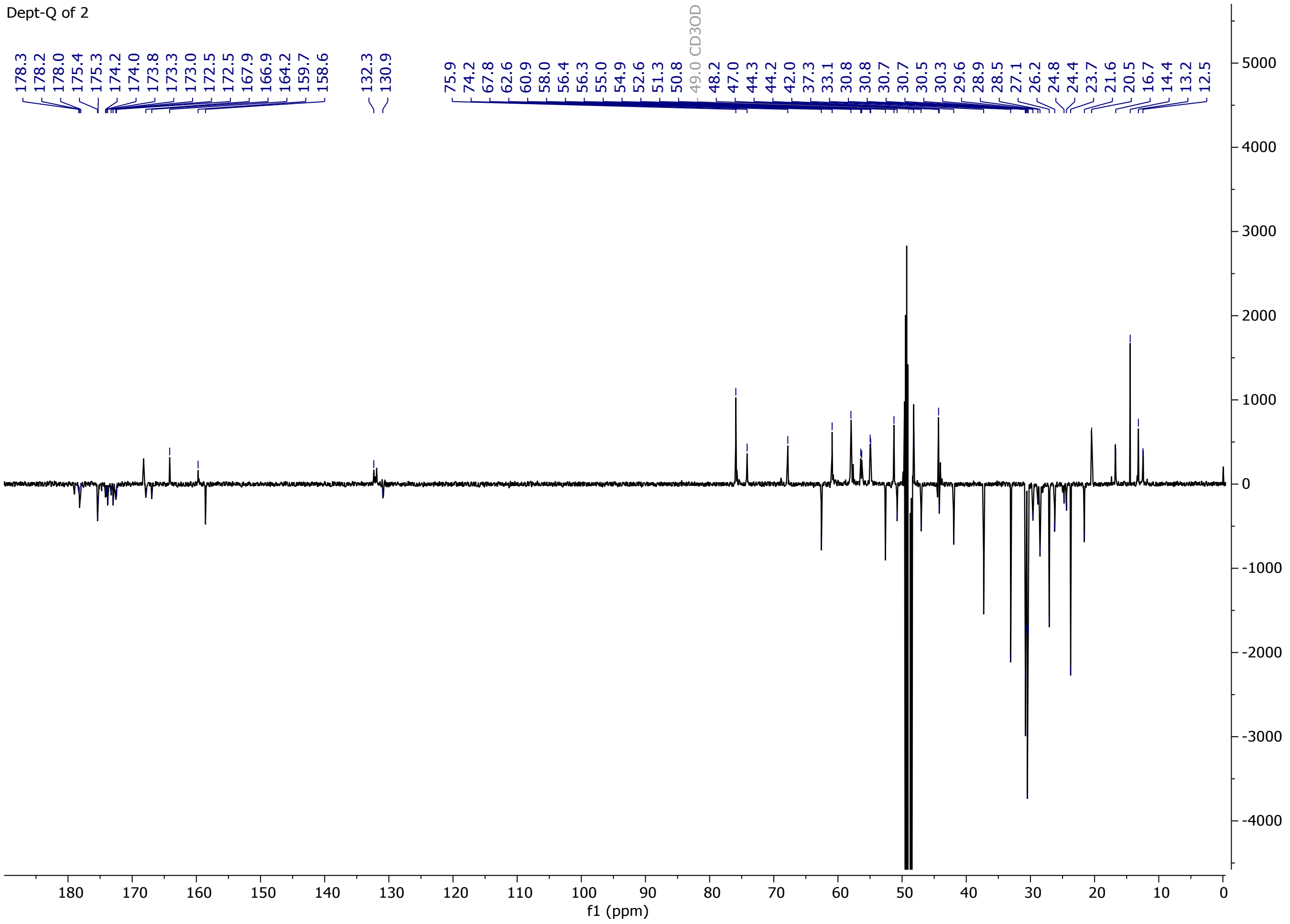

Figure 15: HSQC Spectrum of 2

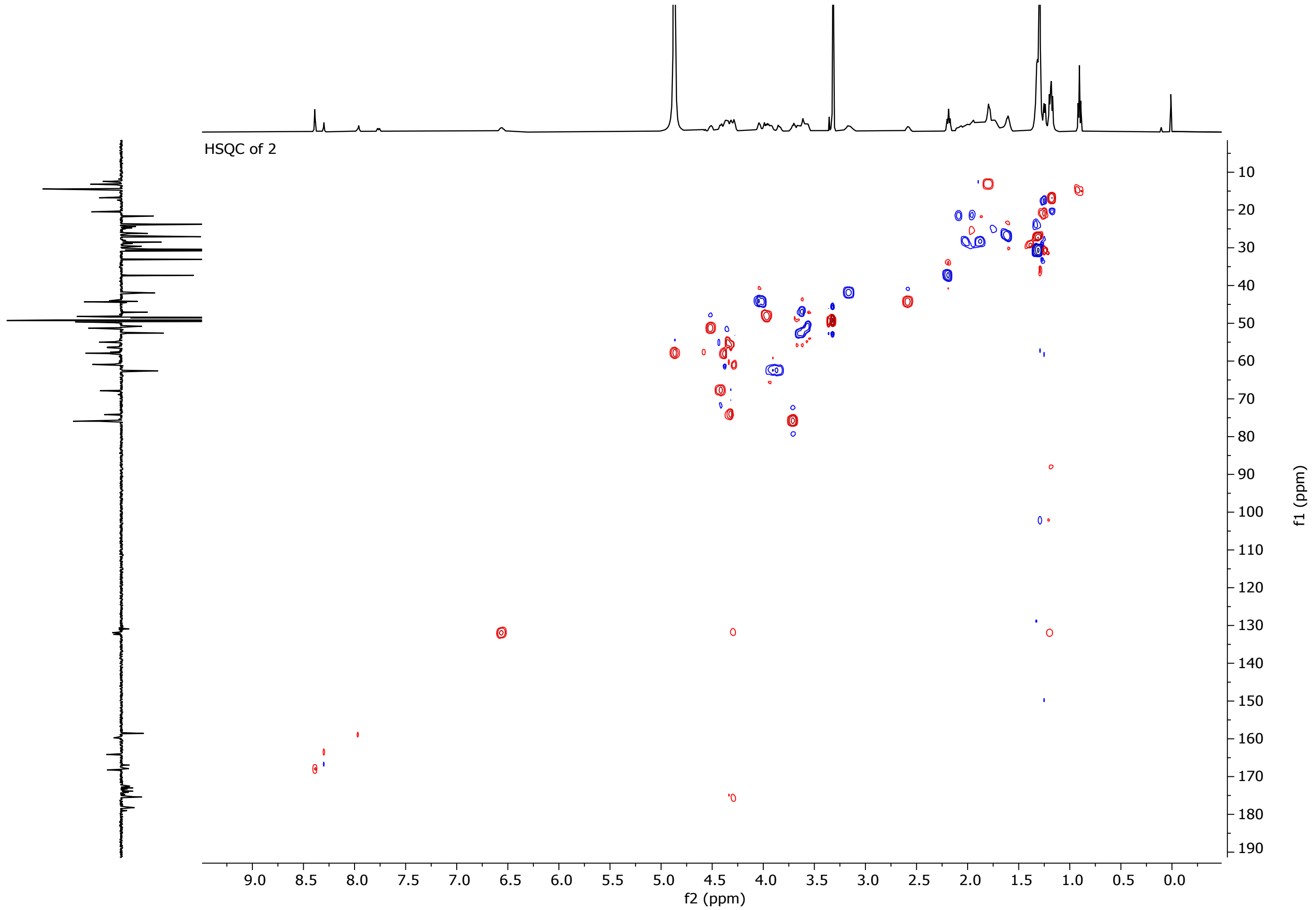

Figure 16: IR Spectrum of 3

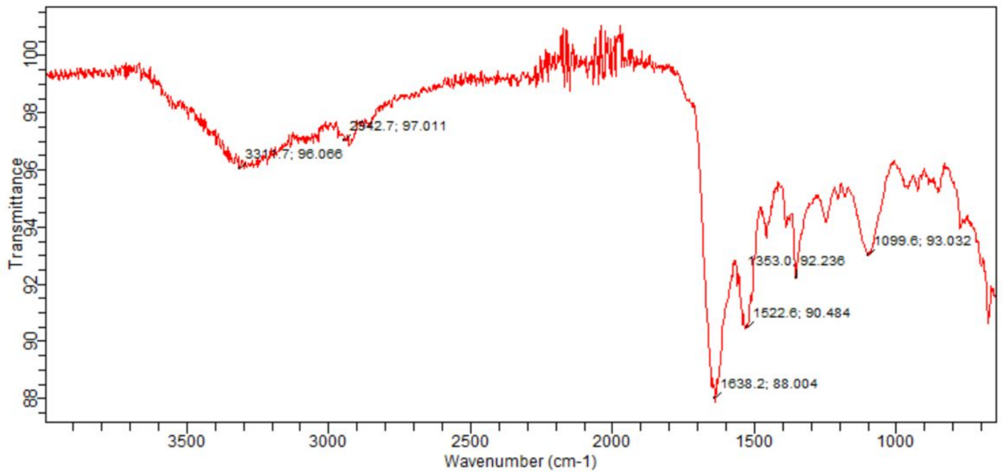

Figure 17: HRMS² Fragment Spectrum of 3

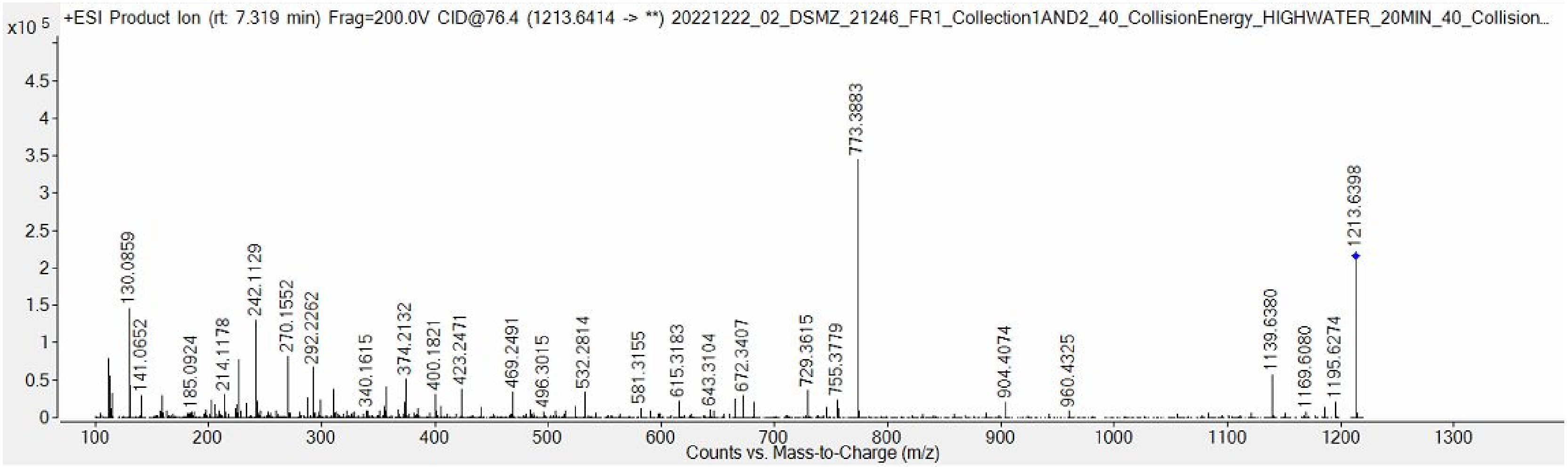

Figure 18: Fragment Structures of 3

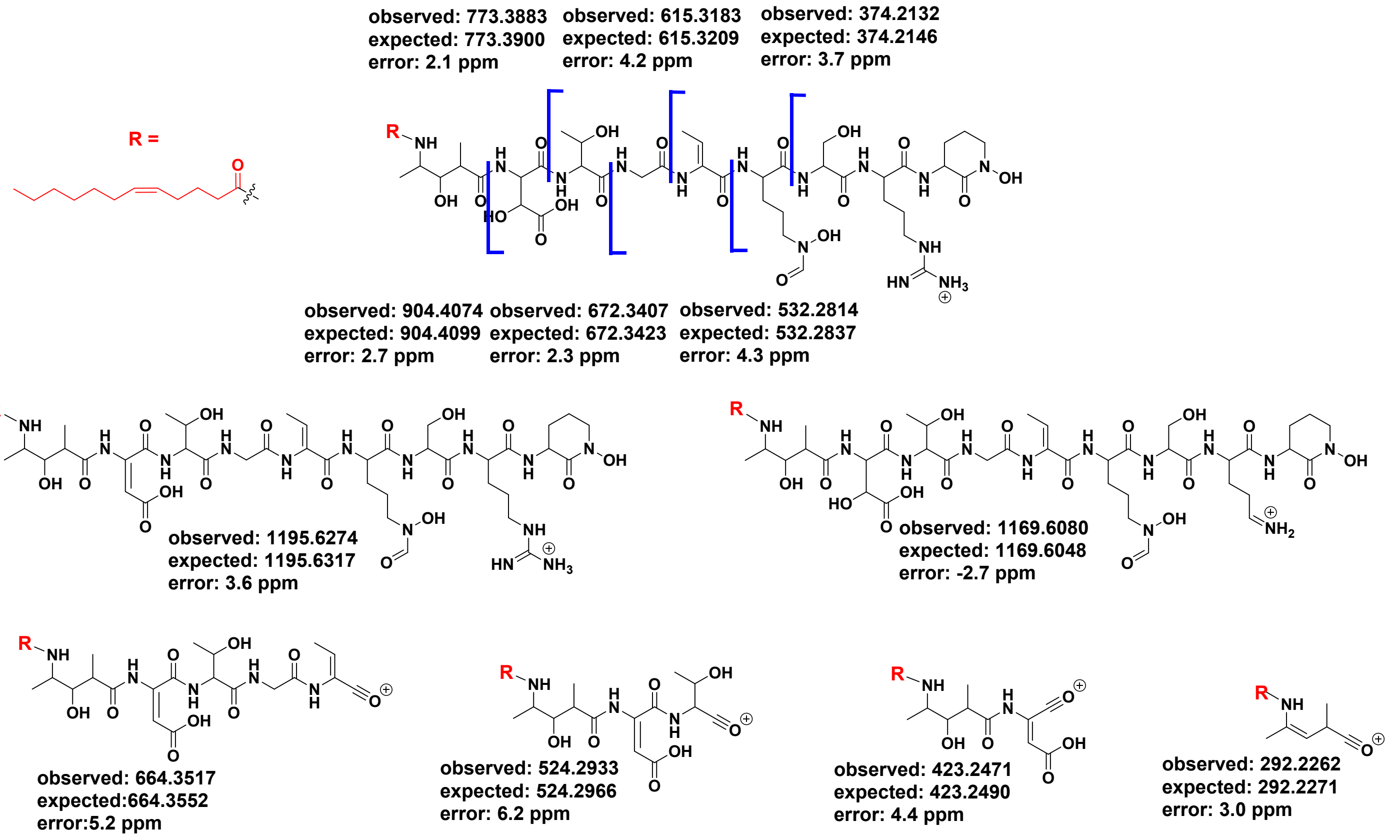

Figure 19: ^{1}H-NMR (500 MHz, CD_3OD) Spectrum of 3

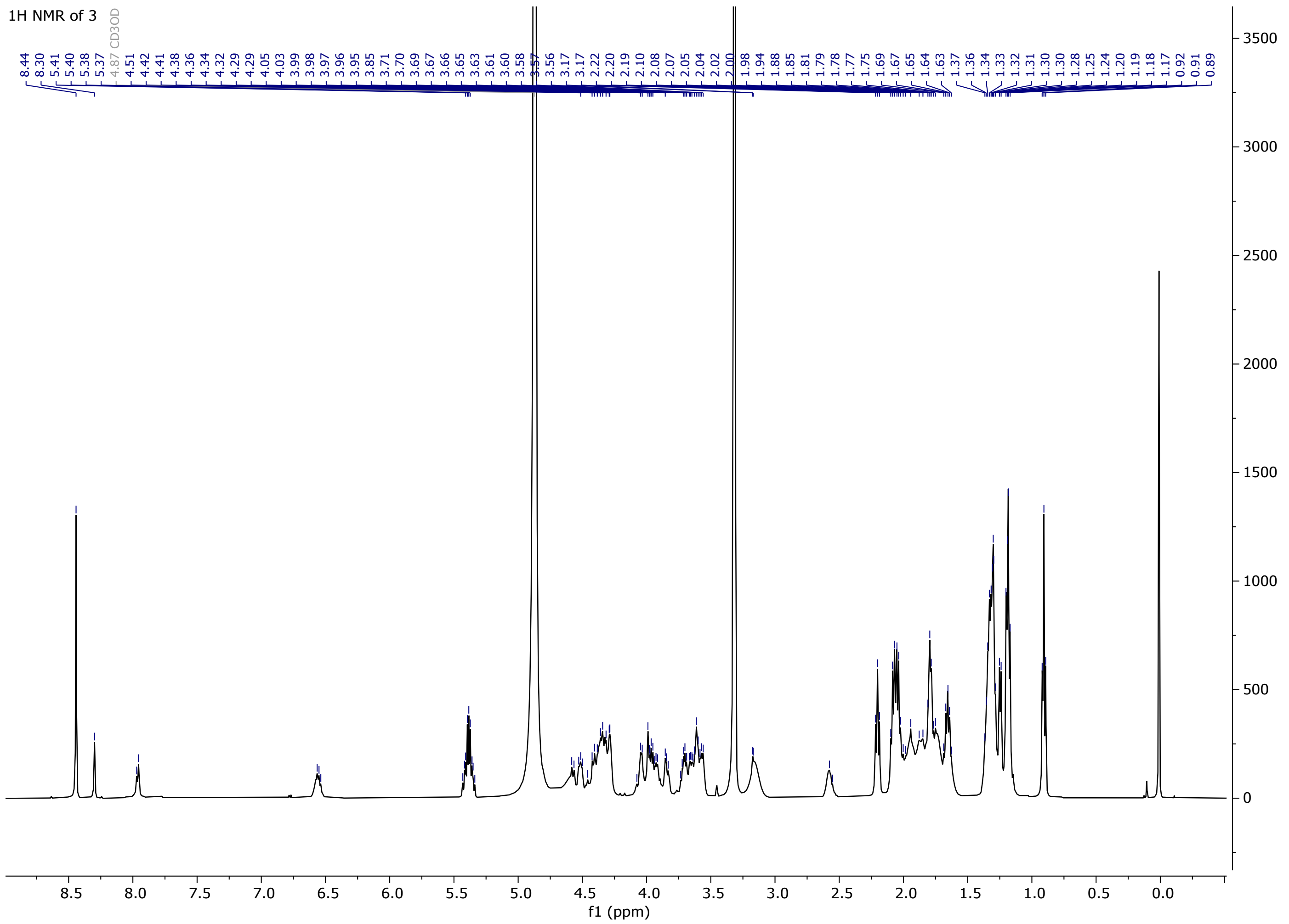

Figure 20: Dept-Q ^{13}C-NMR (125 MHz, CD_3OD) Spectrum of 3

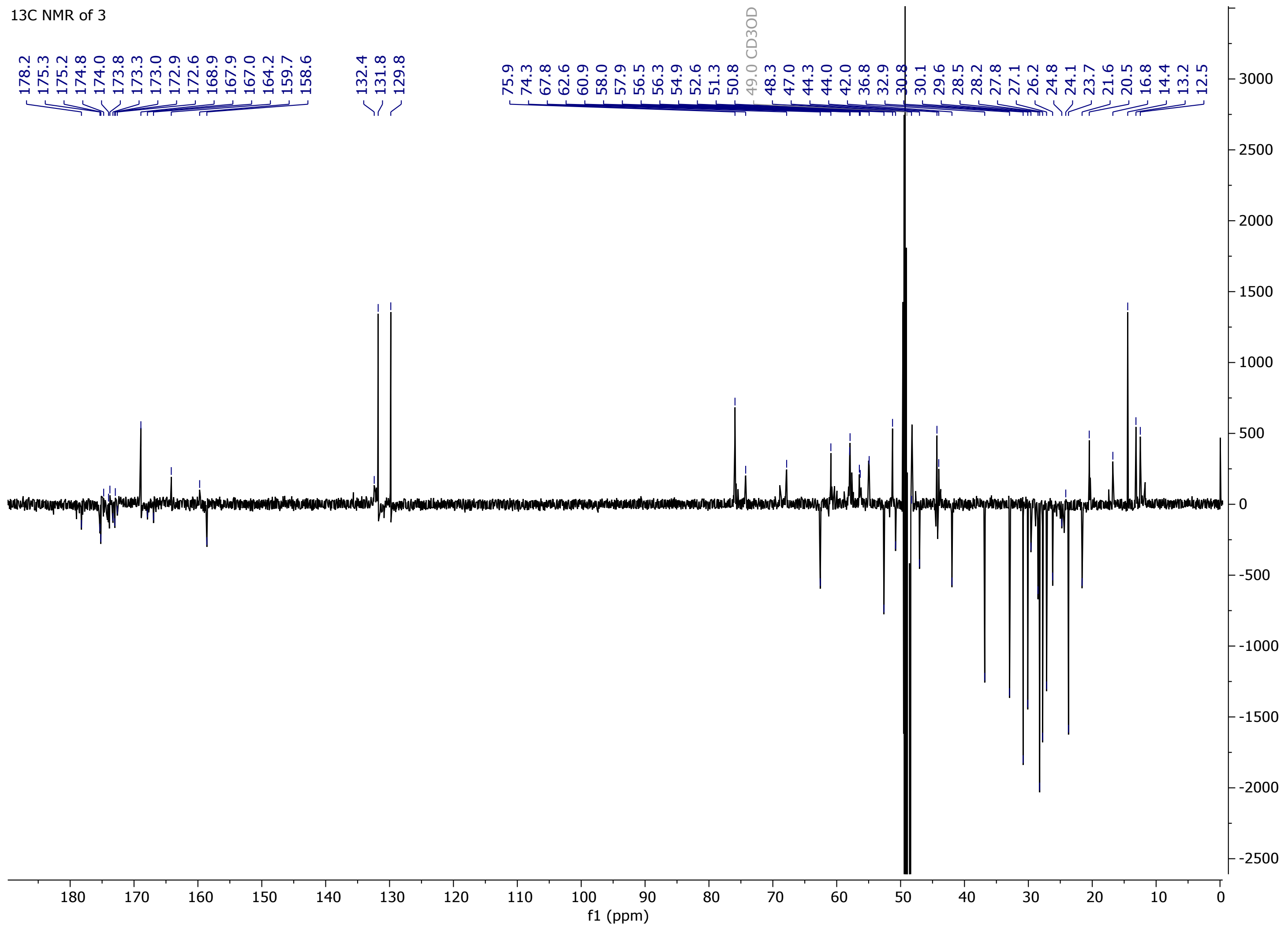

Figure 21: HSQC Spectrum of 3

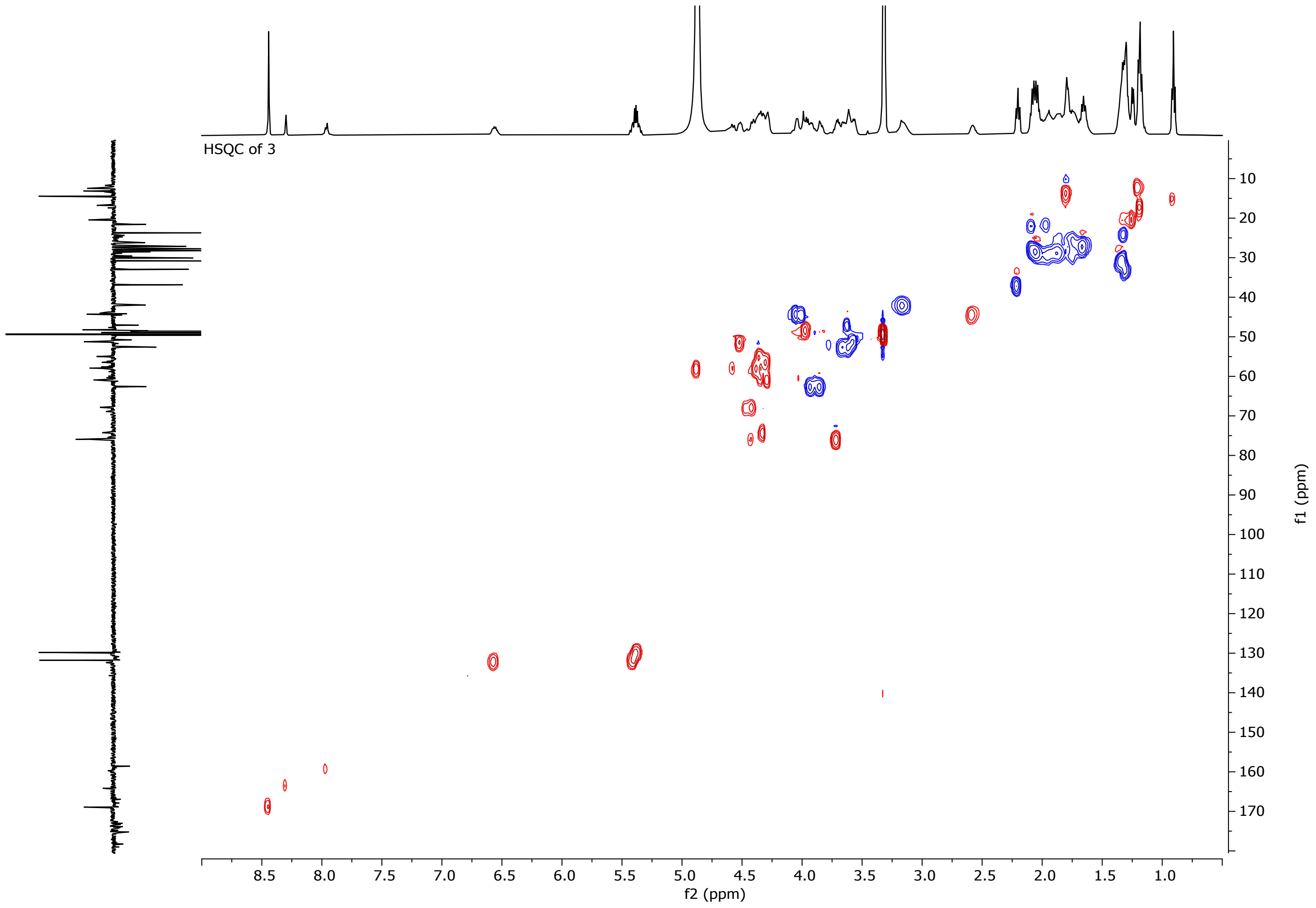

Figure 22: HMBC Spectrum of 3

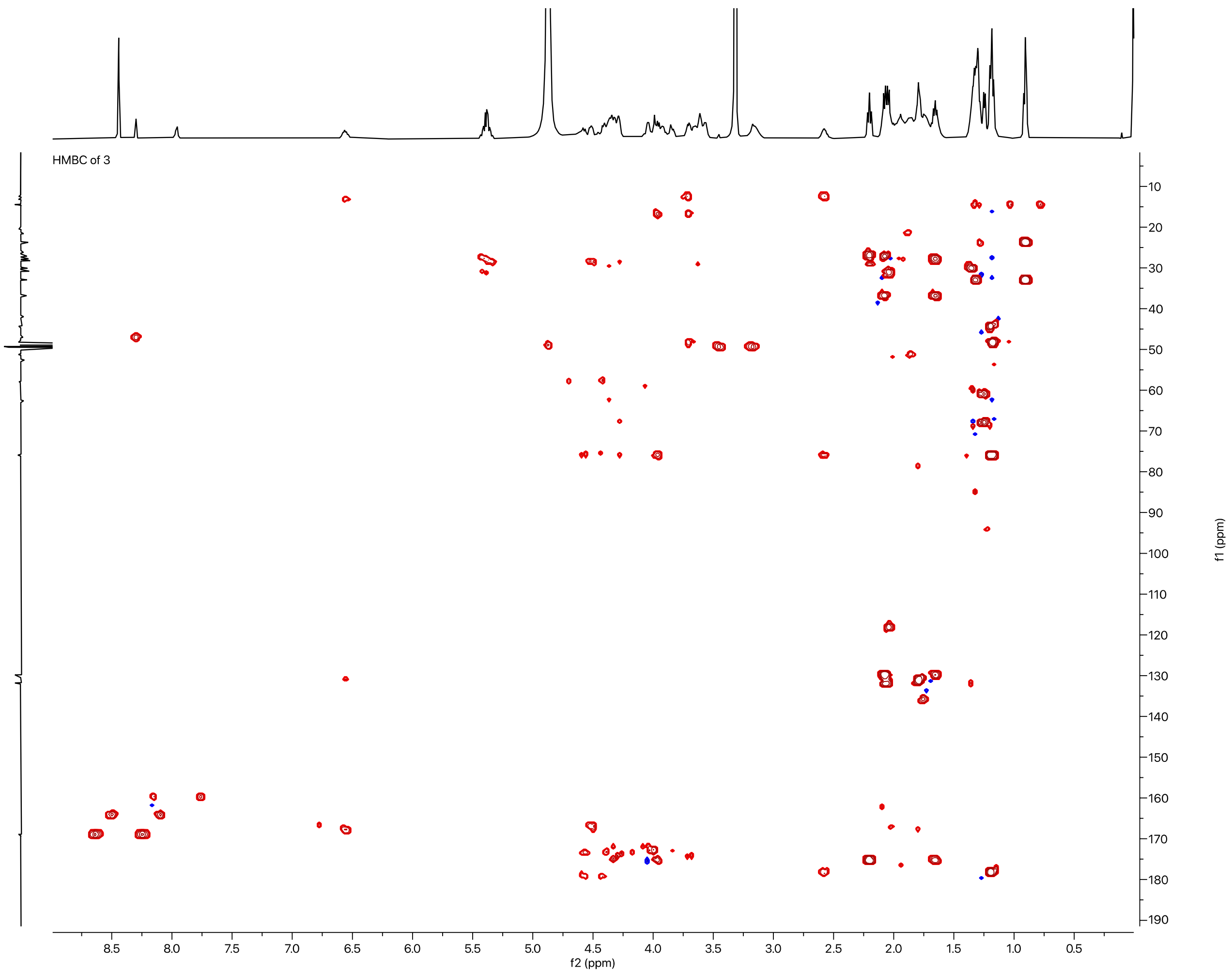

Figure 23: ^{1}H-^{1}H TOCSY Spectrum of 3

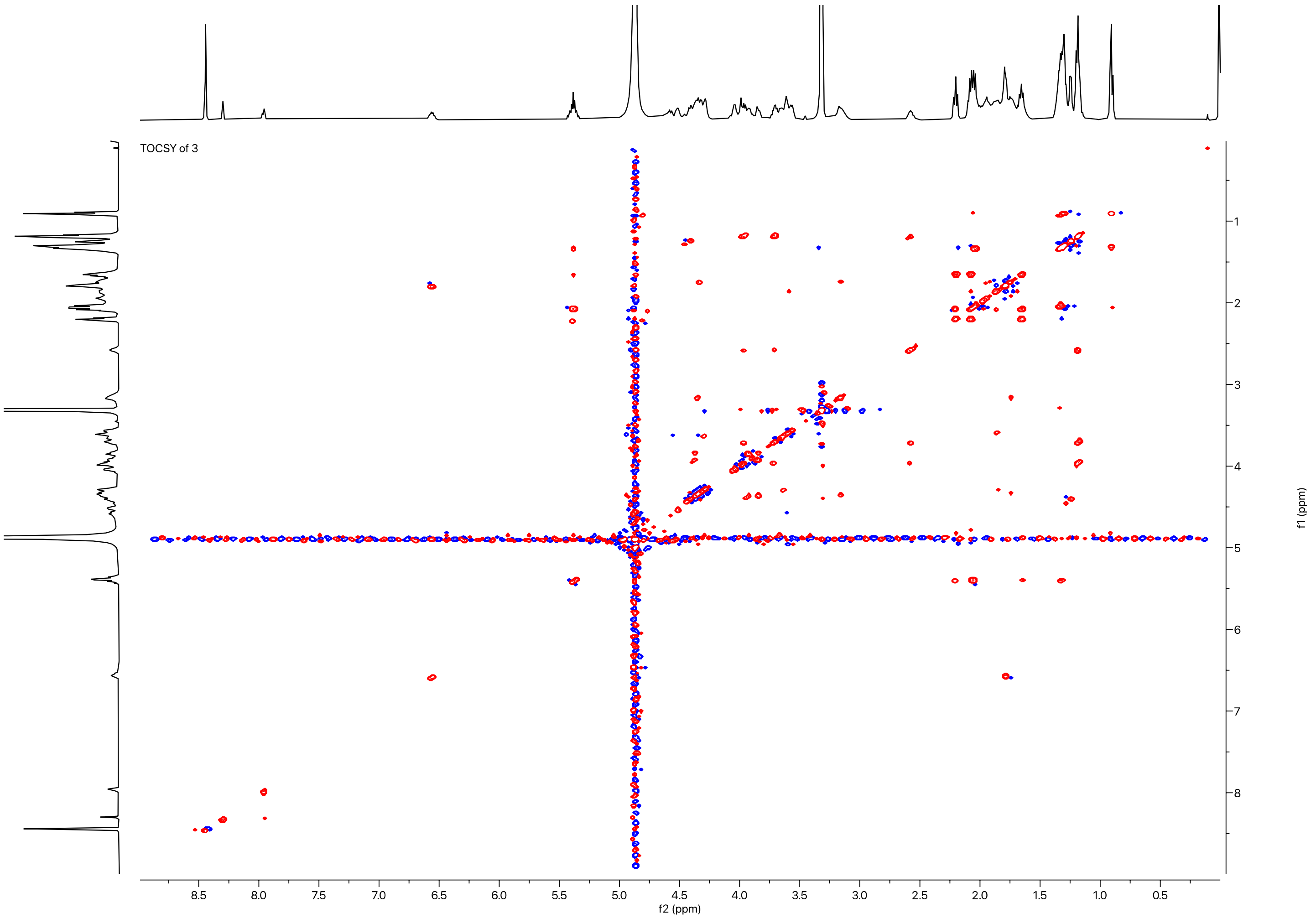

Figure 24: IR Spectrum of 4

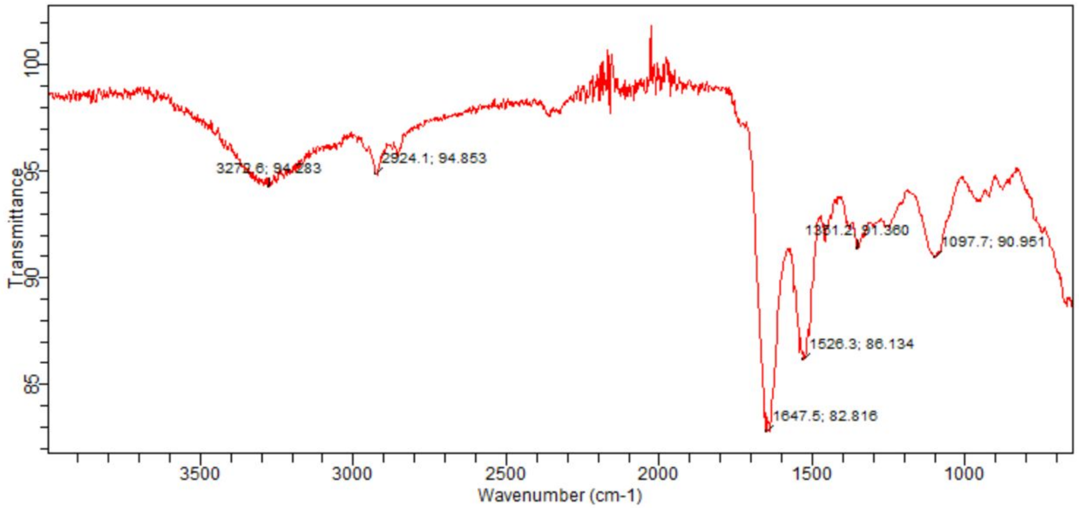

Figure 25: HRMS² Fragment Spectrum of 4

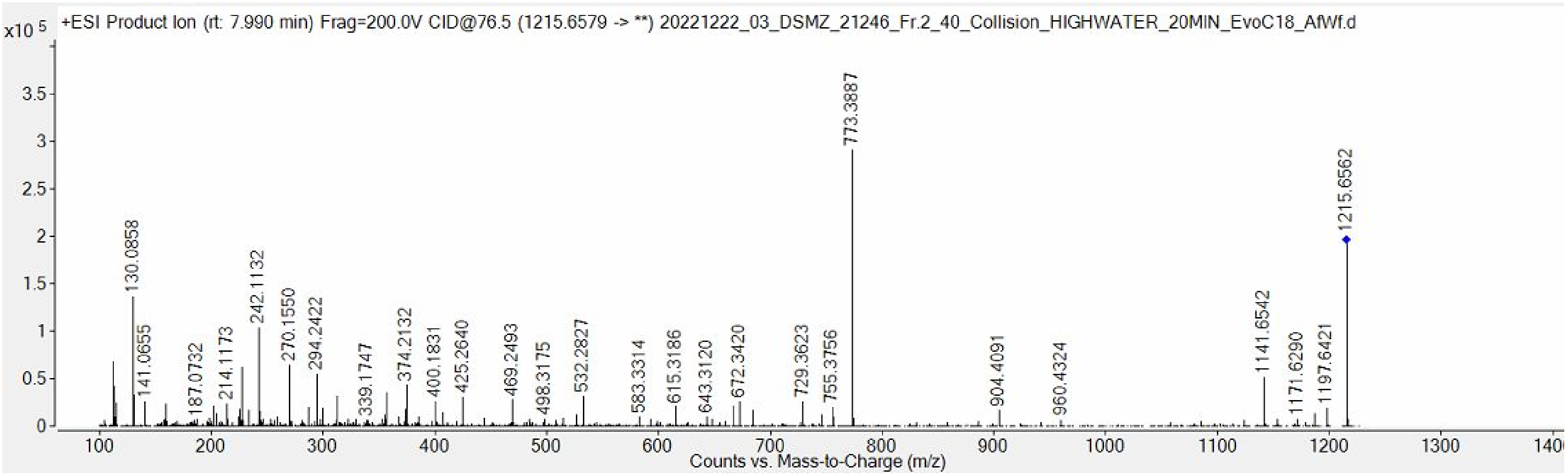

Figure 26: Fragment Structures of 4

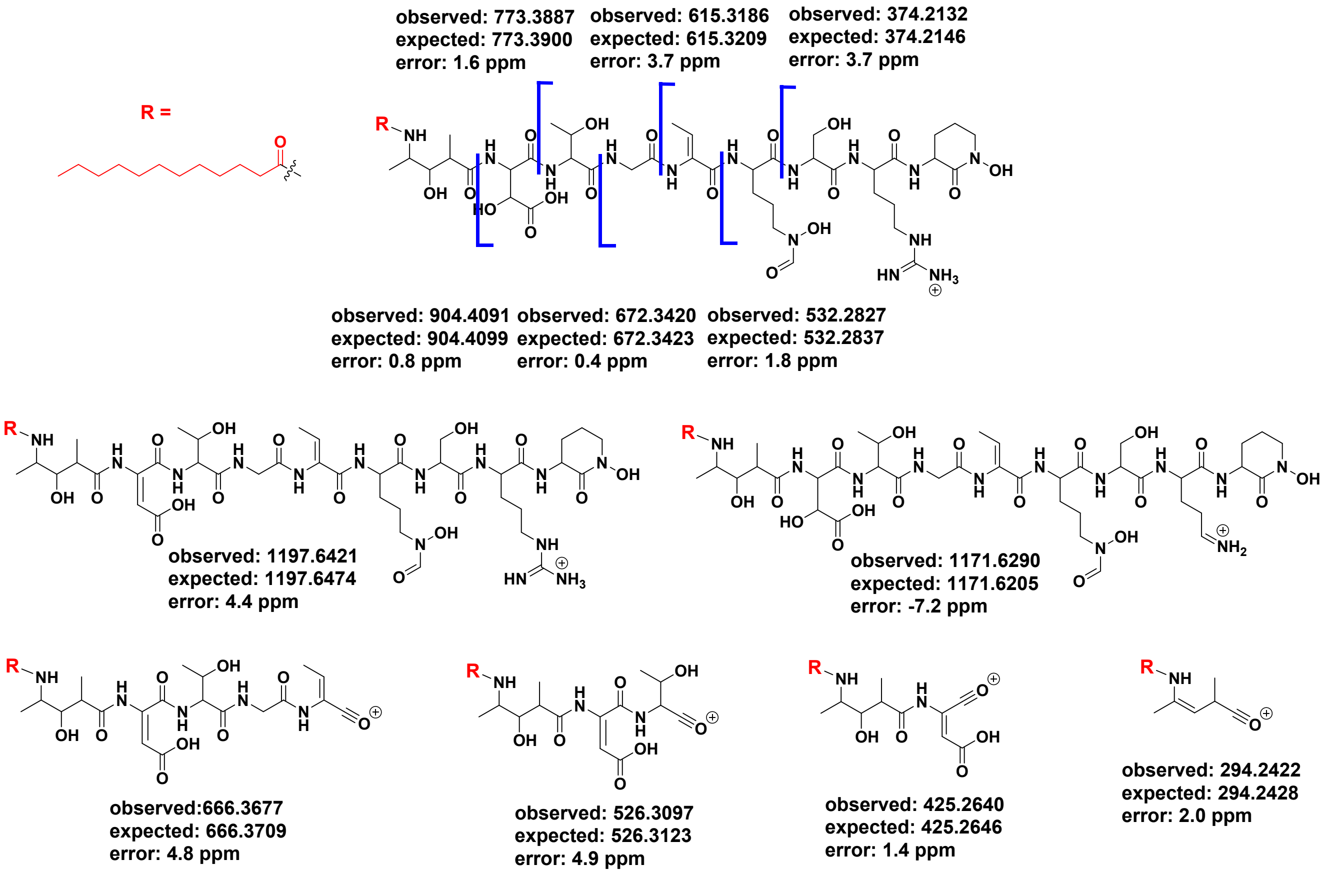

Figure 27: ^{1}H-NMR (500 MHz, CD_3OD) Spectrum of 4

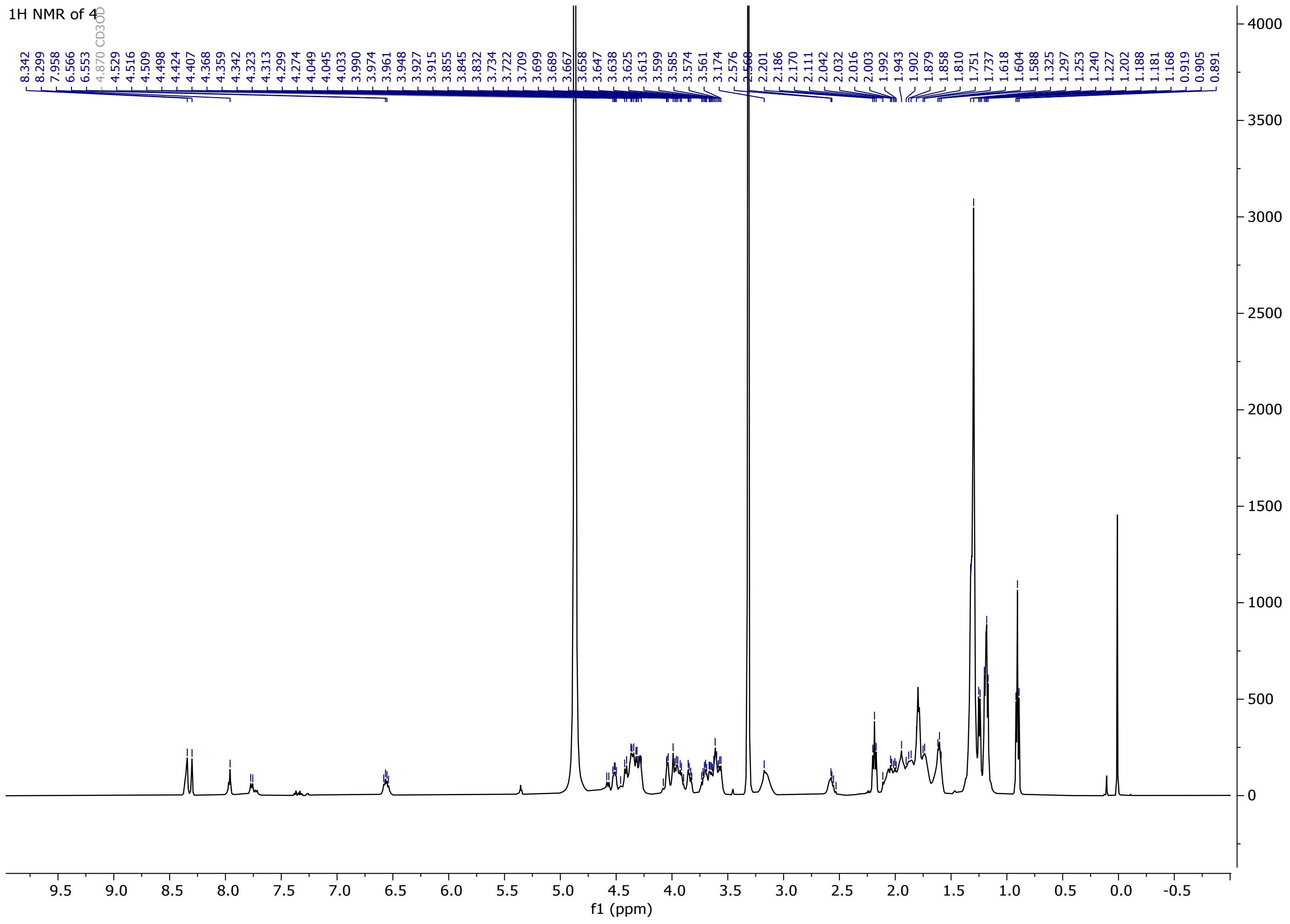

Figure 28: Dept-Q ^{13}C-NMR (125 MHz, CD_3OD) Spectrum of 4

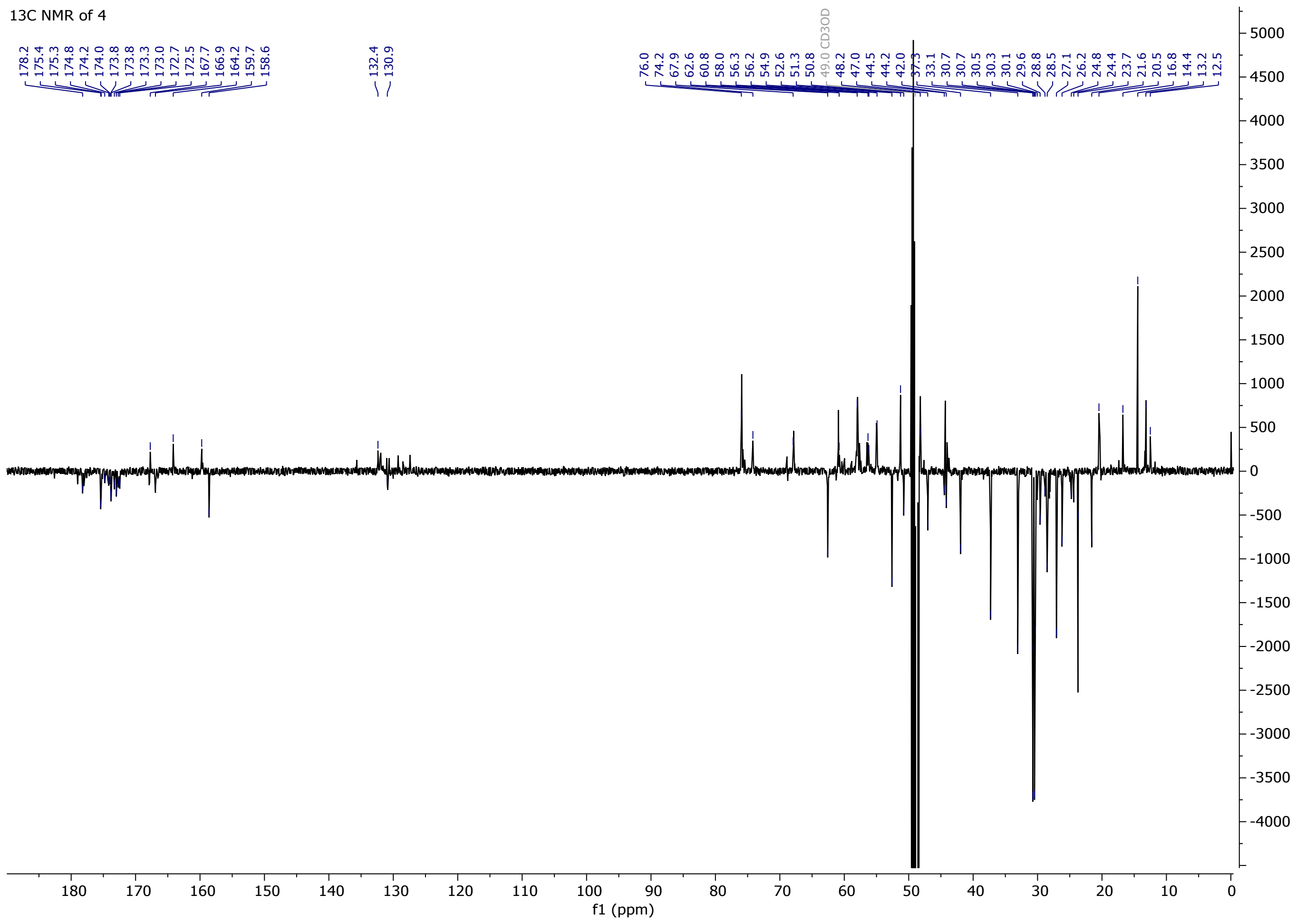

Figure 29: HSQC Spectrum of 4

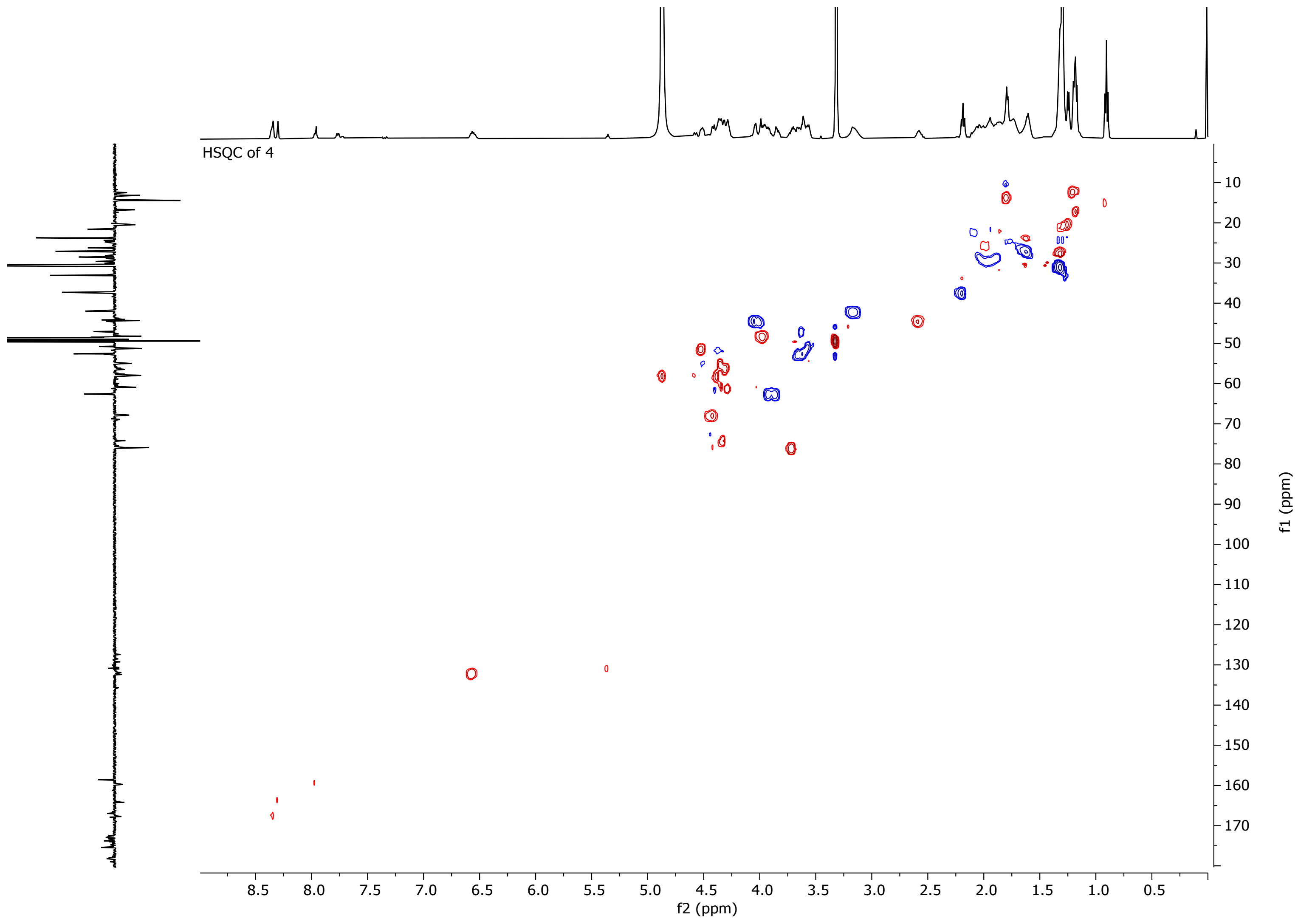

Figure S30: Key TOCSY and HMBC Correlations of Compounds 1 and 3

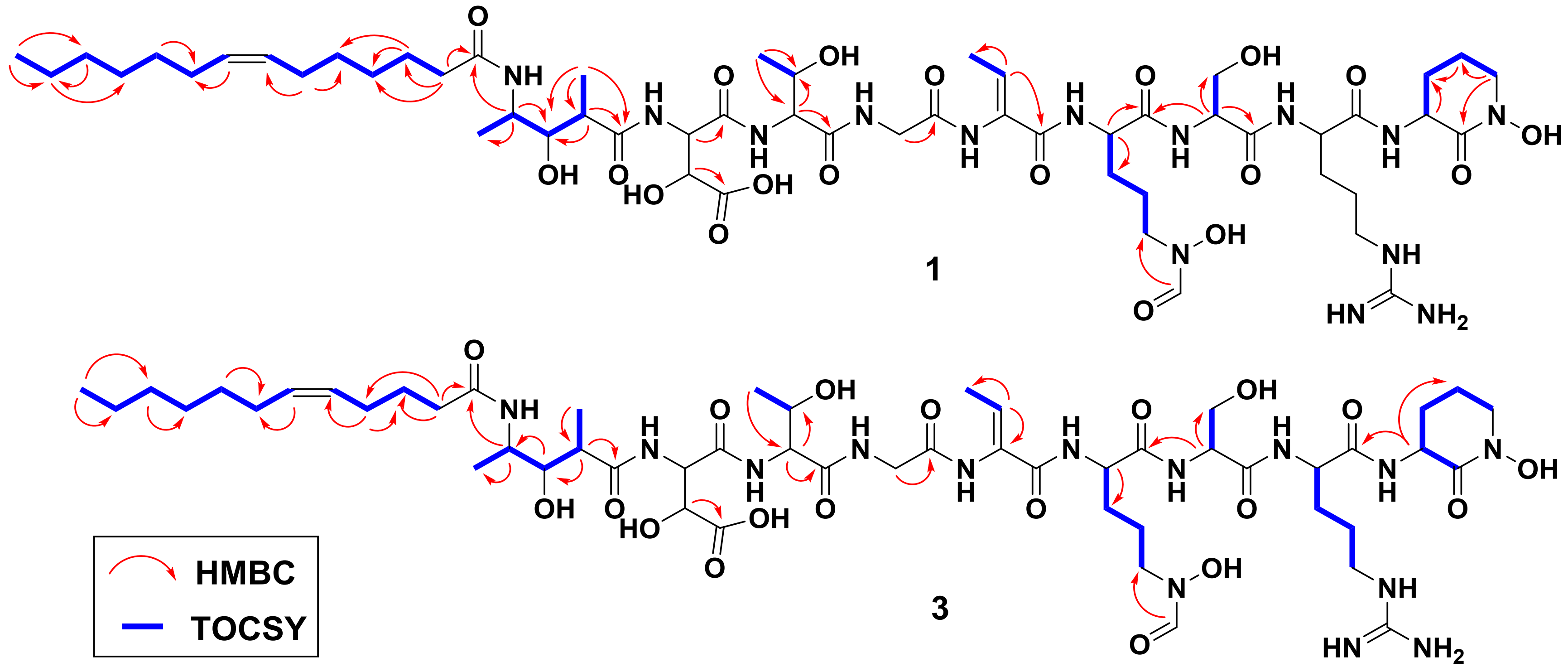

Figure S31: LCMS Chromatograms from the Marfey's Analysis of the Threonine Residue.

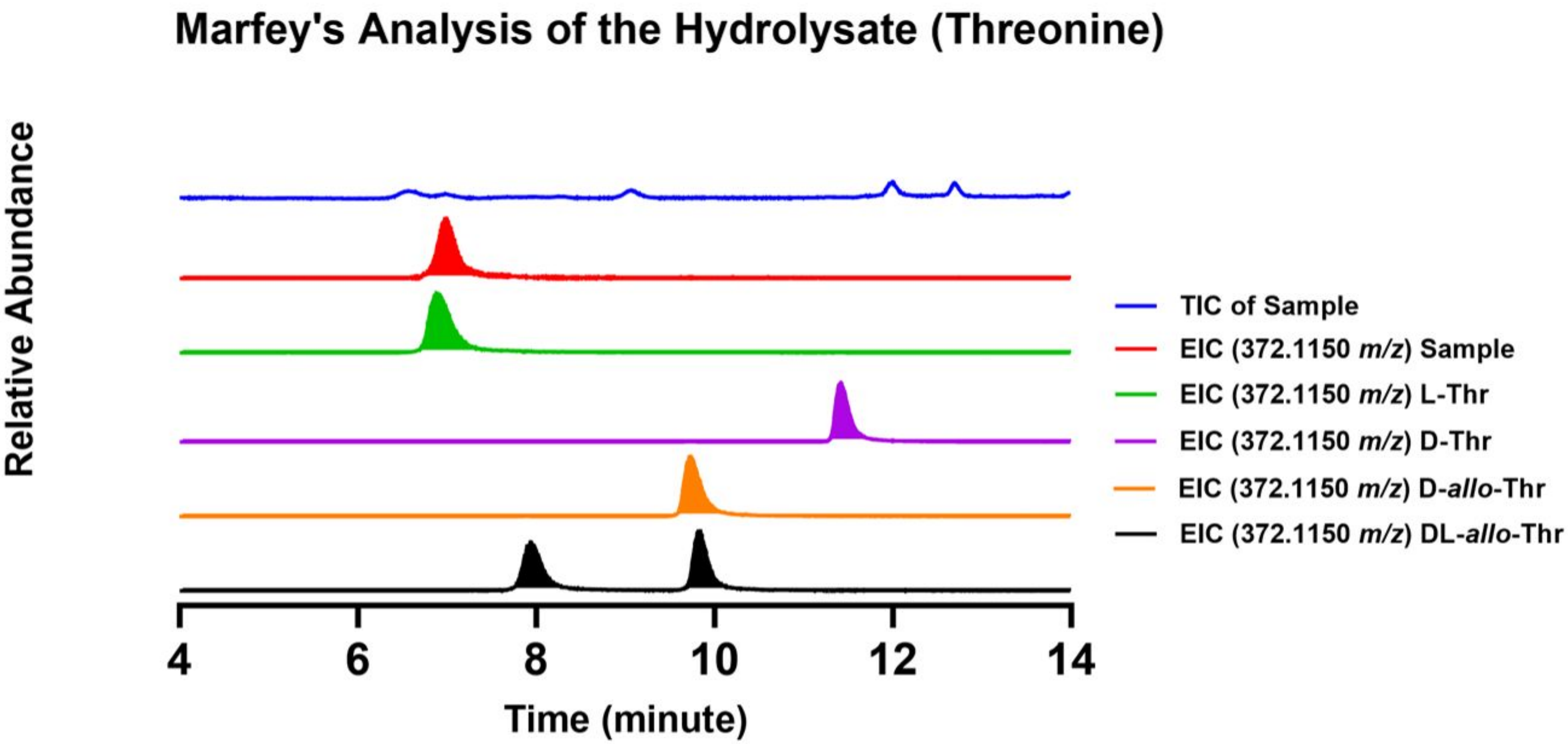

The LCMS Total Ion Chromatogram (TIC) for the hydrolysate of **1** (**Blue**) and the Extracted Ion Chromatogram (EIC) for the Marfey's labeled amino acids in both the sample (**Red**) and the Threonine (Thr) standards (L-Thr as **Green**, D-Thr as **Purple**, D-*allo*-Thr as **Orange**, and D/L-*allo*-Thr as **Black**).

Figure S32: LCMS Chromatograms from the Marfey's Analysis of the Serine Residue.

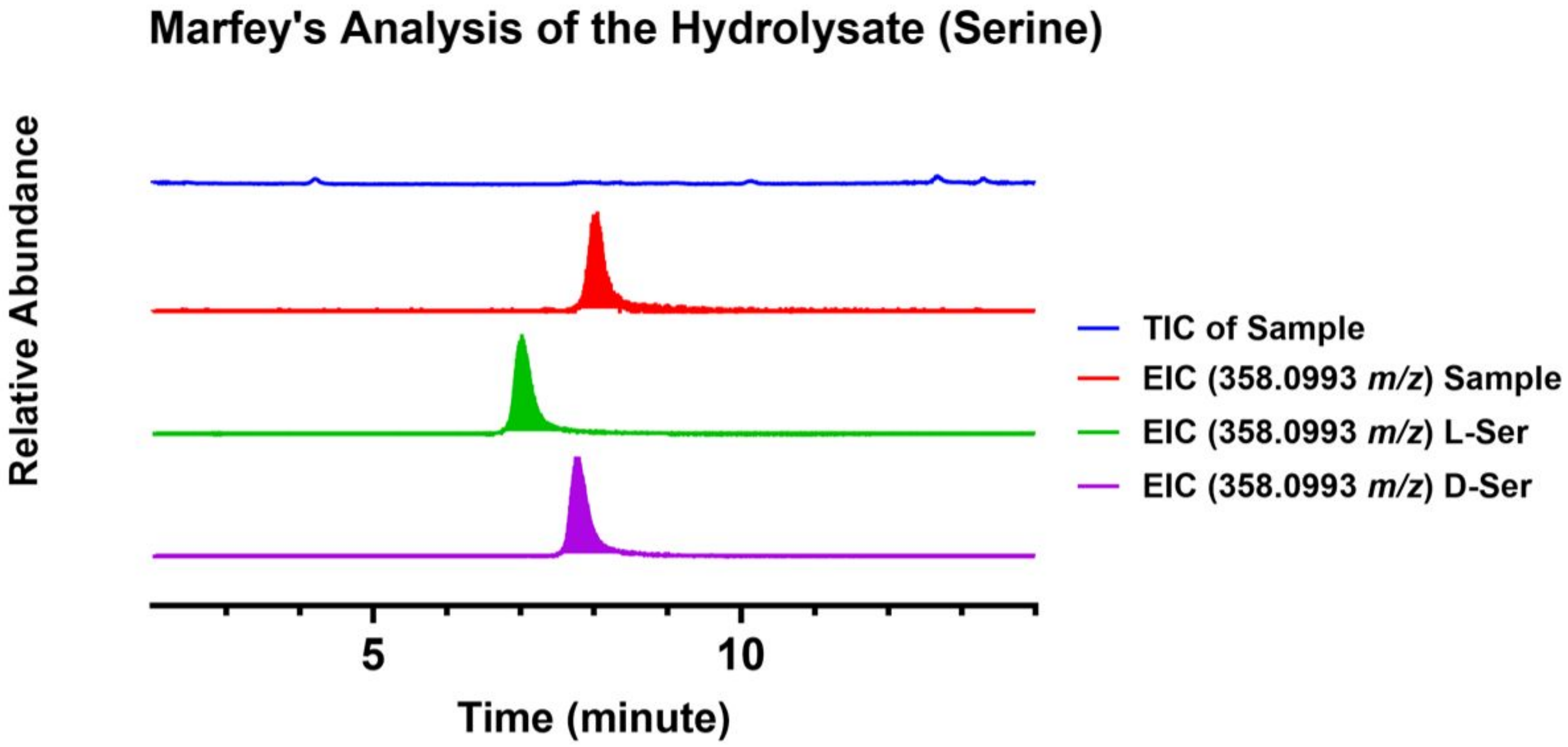

The LCMS Total Ion Chromatogram (TIC) for the hydrolysate of **1** (**Blue**) and the Extracted Ion Chromatogram (EIC) for the Marfey's labeled amino acids in both the sample (**Red**) and the Serine (Ser) standards (L-Ser as **Green**, D-Ser as **Purple**).

Figure S33: LCMS Chromatograms from the Marfey's Analysis of the Ornithine Residues.

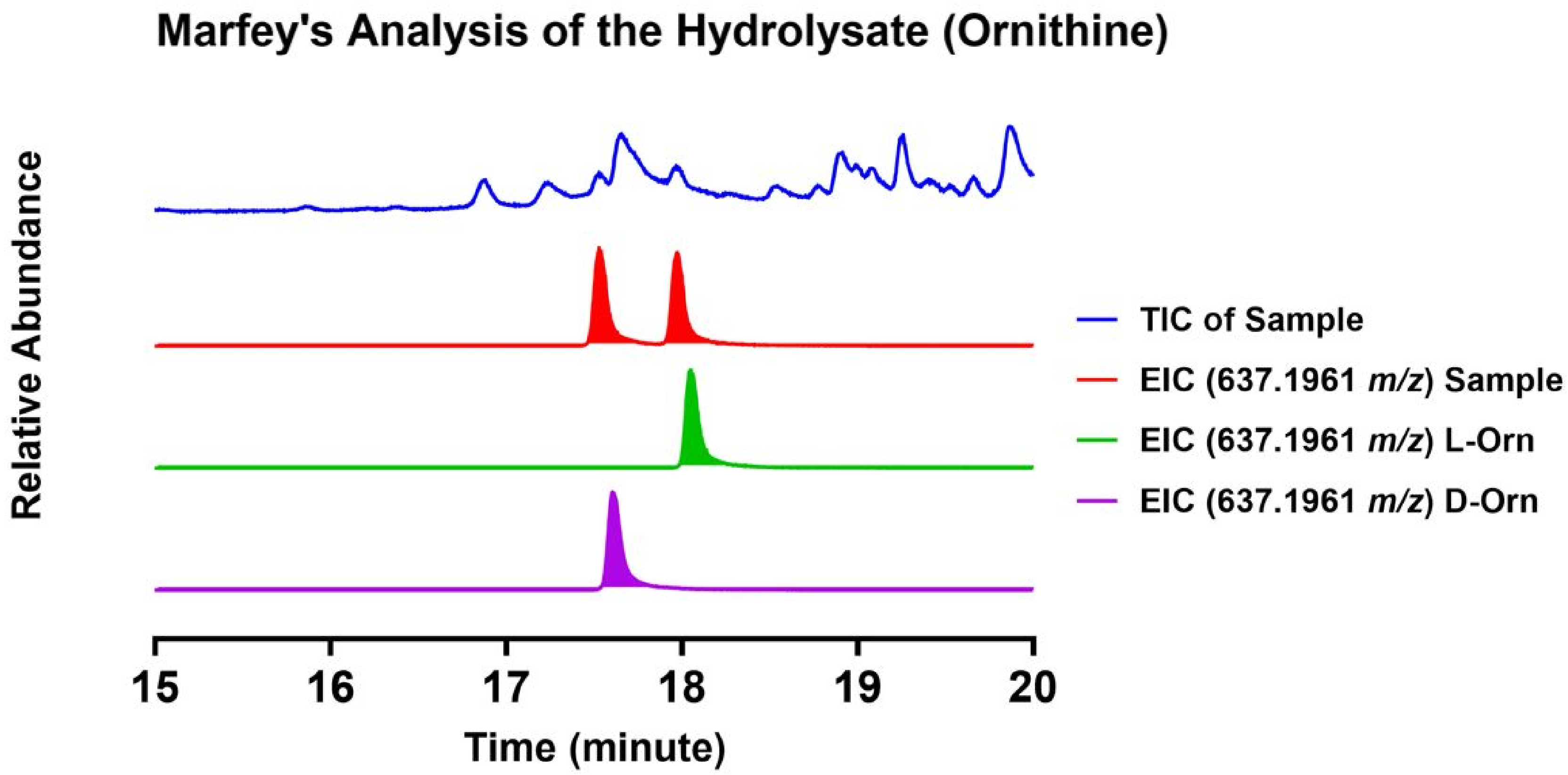

The LCMS Total Ion Chromatogram (TIC) for the hydrolysate of **1** (**Blue**) and the Extracted Ion Chromatogram (EIC) for the Marfey's labeled amino acids in both the sample (**Red**) and the Ornithine (Orn) standards (L-Orn as **Green**, D-Orn as **Purple**).

Figure S34: LCMS Chromatograms from the Marfey's Analysis of the Arginine Residues.

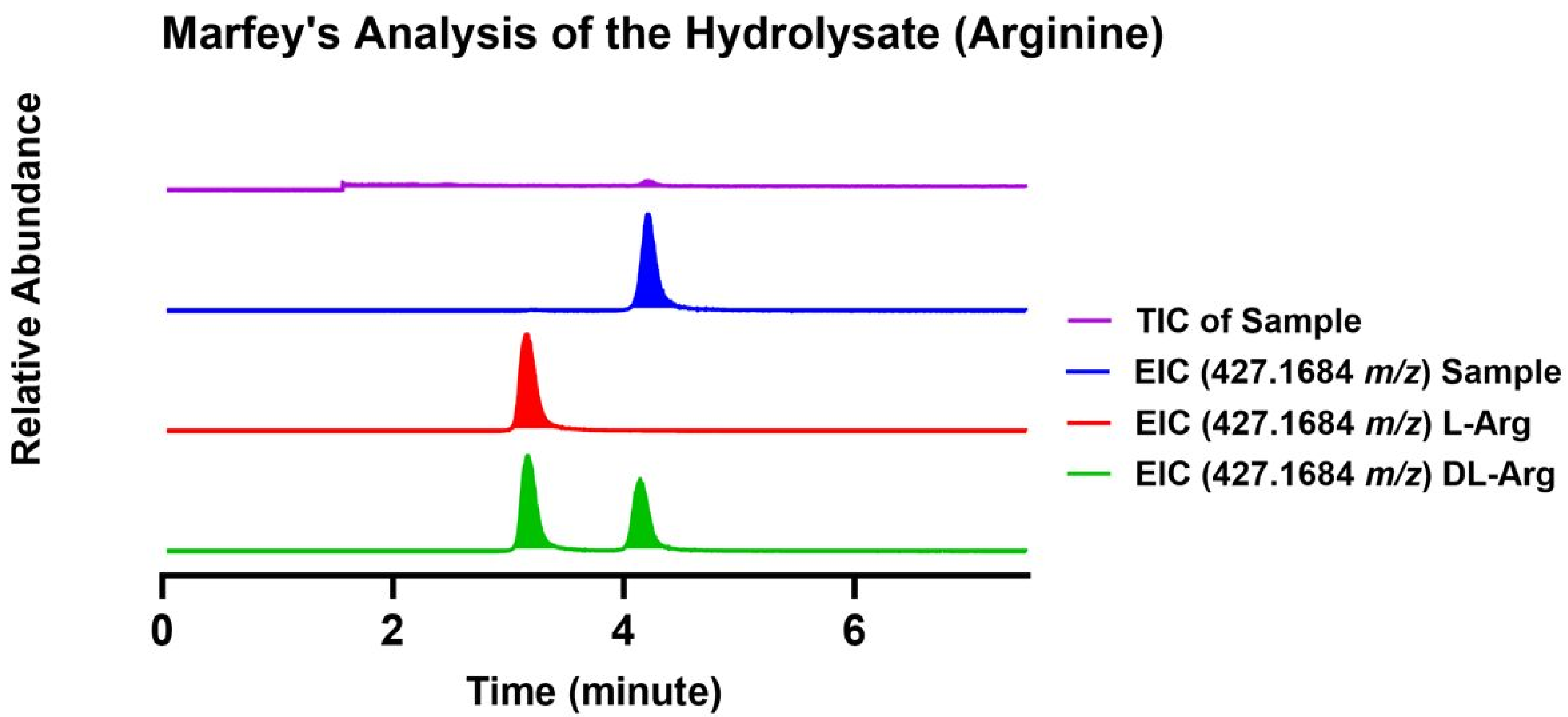

The LCMS Total Ion Chromatogram (TIC) for the hydrolysate of **1** (**Purple**) and the Extracted Ion Chromatogram (EIC) for the Marfey's labeled amino acids in both the sample (**Blue**) and the Arginine (Arg) standards (L-Arg as **Red**, D/L-Arg as **Green**).

Table S1: Analysis of the Delftibactin Biosynthetic Gene Cluster

Relative position in BGC (bp)	Gene	RAST Predicted Function	Strand	Amino Acids	% Pairwise Identity to *D. acidovorans* SPH-1 BGC*
1-363	*delA*	MbtH-like NRPS chaperone	-	120	92.6
369-1,112	*delB*	Thioesterase involved in non-ribosomal peptide biosynthesis	-	247	98.0
1,090-1,779	*delC*	4'-phosphopantetheinyl transferase	-	229	96.9
1,776-2,765	*delD*	SyrP-like protein	-	329	99.7
2,786-8,146	*delE*	Non-ribosomal peptide synthetase modules	-	1,786	97.3
8,146-12,813	*delF*	Polyketide synthase modules and related proteins	-	1,555	96.0
12,824-22,744	*delG*	Siderophore biosynthesis non-ribosomal peptide synthetase modules	-	3,306	96.9
22,744-41,340	*delH*	Siderophore biosynthesis non-ribosomal peptide synthetase modules	-	6,198	97.0
41,526-43,925	*delI*	Outer membrane ferripyoverdine receptor FpvA, TonB-dependent	-	799	98.5
44,002-45,036	*delJ*	Putative transmembrane sensor	+	344	91.9
45,049-45,648	*delK*	RNA polymerase ECF-type sigma factor	+	199	96.5
45,863-47,161	*delL*	L-lysine 6-monooxygenase (Lysine N(6)-hydroxylase) protein	+	432	98.6
47,173-48,393	*delM*	hypothetical protein	+	406	95.8
48,438-49,403	*delN*	Esterase/lipase	+	321	96.0
49,651-51,372	*delO*	ABC-type siderophore export system, fused ATPase and permease components	-	573	97.9
51,394-52,236	*delP*	Pyoverdine synthetase PvdF, N5-hydroxyornithine formyltransferase	-	280	97.5

*Reference amino acid sequences from MIBiG repository: BGC0000984, aligned in Geneious Prime 2023.0.4 with MUSCLE v5.1 Super5 alignment tool.

Figure S35: **Multiple Sequence Alignment of Amino Acids for Delftibactin BGC Peptide Carrier Protein (PCP) Domains.**

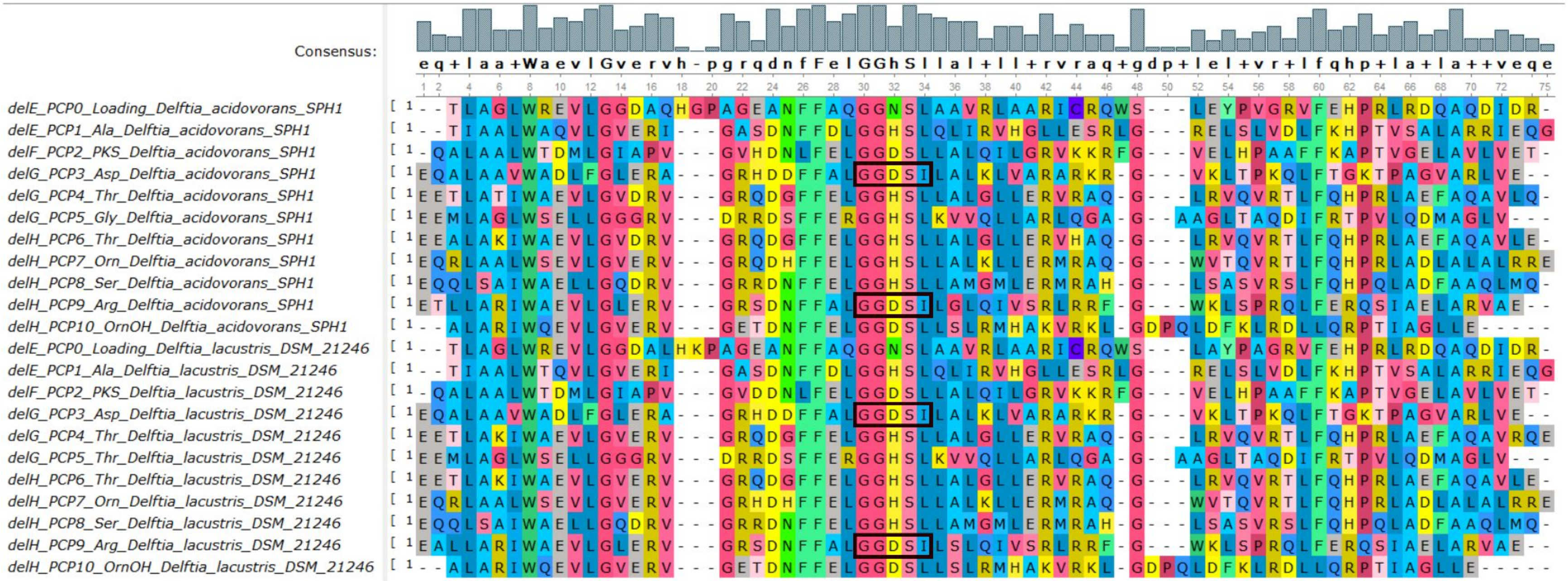

The highlighted (**Black**) "GGDSI" motifs which were reported in the original biosynthetic gene cluster,[1] are highly conserved in thiolation (PCP) domains followed by an epimerization domain.[2,3] The shared conservation of these motifs, note delH at PCP domain nine which is followed by an epimerization domain, between these clusters is supportive of the notion that the delftibactins (discovered so far) share the same epimerization at this position in the peptidic chain. In addition, Reitz and coworkers prior investigations into the actions of DelD argued that this domain is essential to that stand-alone hydroxylase acting to make L-*erythro*-Asp in delftibactin, so the presence of this motif on DelG is consistent with that mechanism and the previously known version of this BGC.[1,3]

Figure S36: **Multiple Sequence Alignment of Amino Acids for Delftibactin BGC Condensation Domains and Reported Dual Condensation (C^D) Domains.**

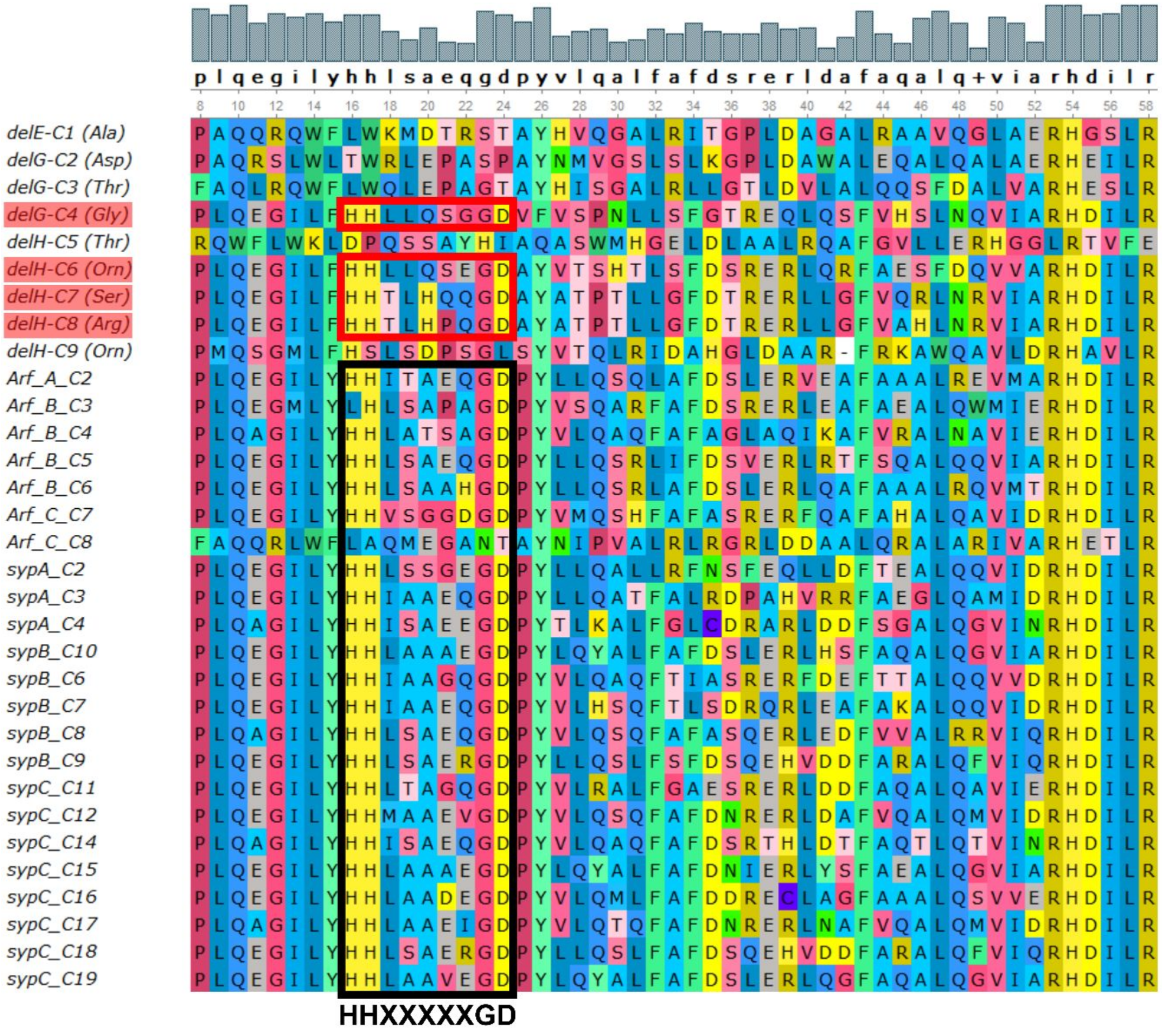

The highlighted (**Black**) "HHXXXXXGD" motif which was reported in C^D domains.[4,5] Four of the condensation domains in our BGC which share the same motif are also highlighted (**Red**).

Figure S37. Interaction of delftibactin C (1) with metals (iron, gold, and copper).

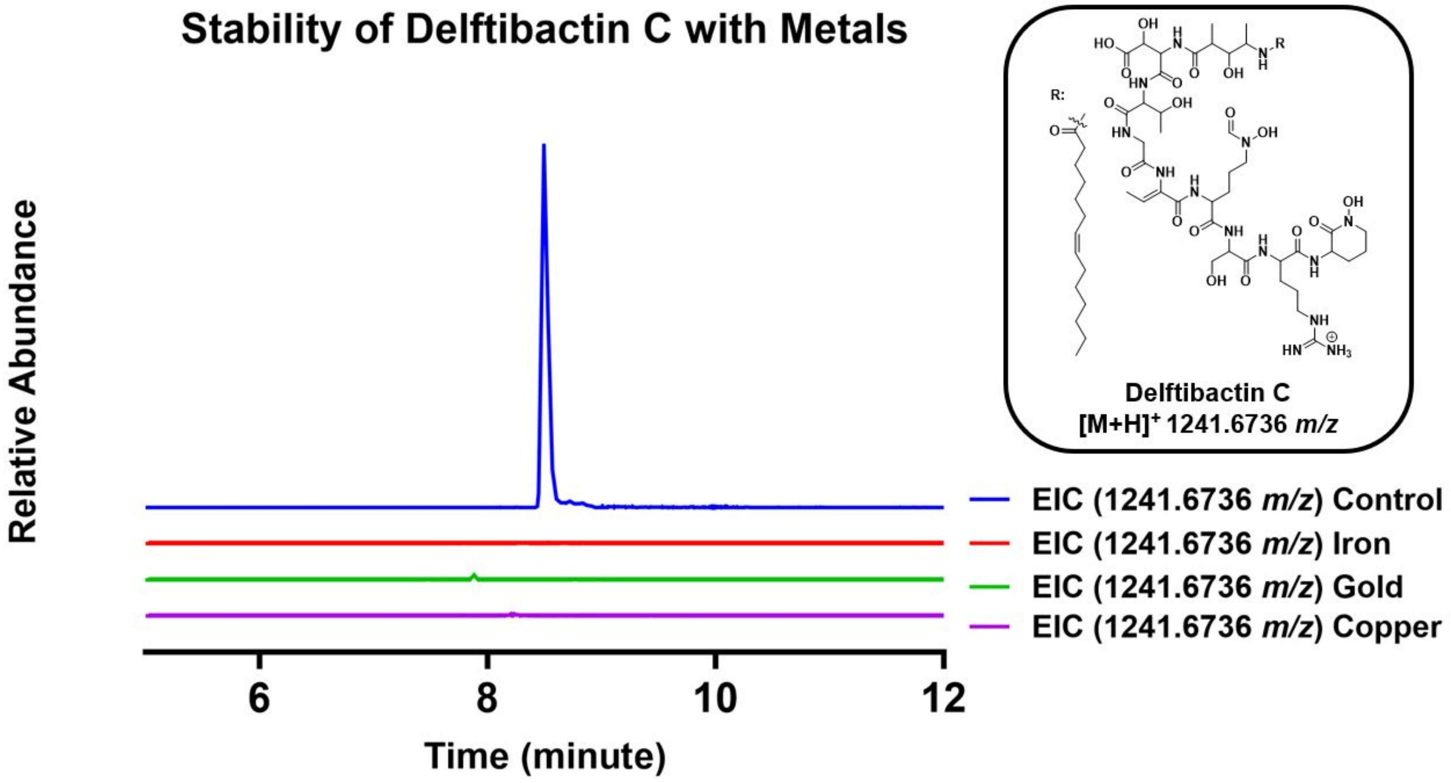

Control group contains delftibactin C in Milli-Q water (**Blue**), while interaction of delftibactin C with iron ($FeCl_3$) in water (**Red**) showed loss of the $[M+H]^+$ mass for a compound-ferric complex (see Figure S37). Delftibactin C reacted with gold ($AuCl_3$) or copper ($CuCl_2$) in water saw the loss of the siderophore for degrative products (**Green** and **Purple** chromatograms, respectively). See Figure S38 for degradation products after reaction with gold.

Figure S38. Formation of an iron adduct of delftibactin C (1) with iron.

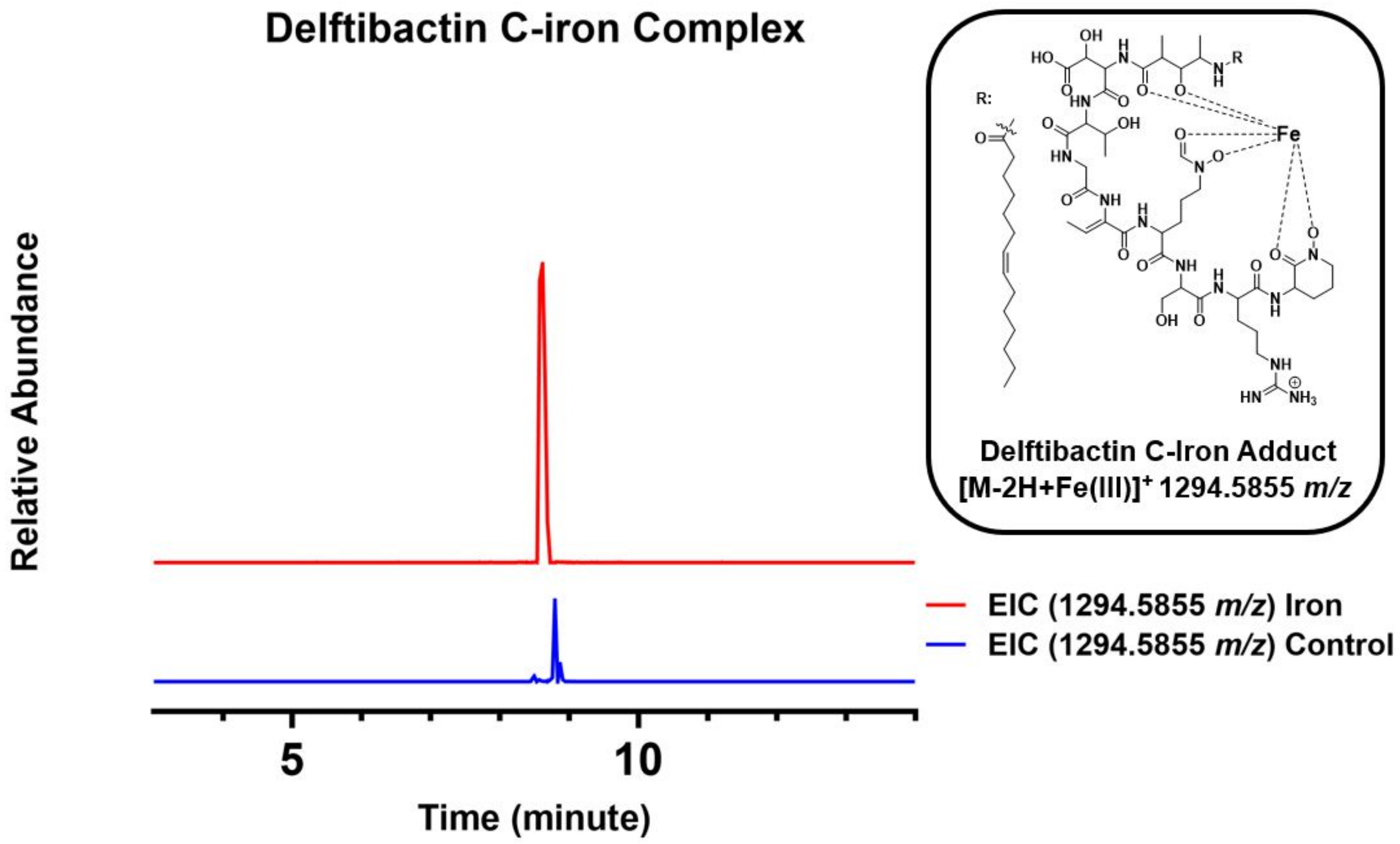

The strong binding of delftibactin C to iron allows us to see the iron adduct in a control without added iron salt (**Blue**). Adding iron ($FeCl_3$) to delftibactin C showed the complete conversion of the delftibactin $[M+H]^+$ ion to a delftibactin C-iron complex (**Red**).

Figure 39. Formation of a gold precipitate and an oxidative degradation product of delftibactin C (1) with gold.

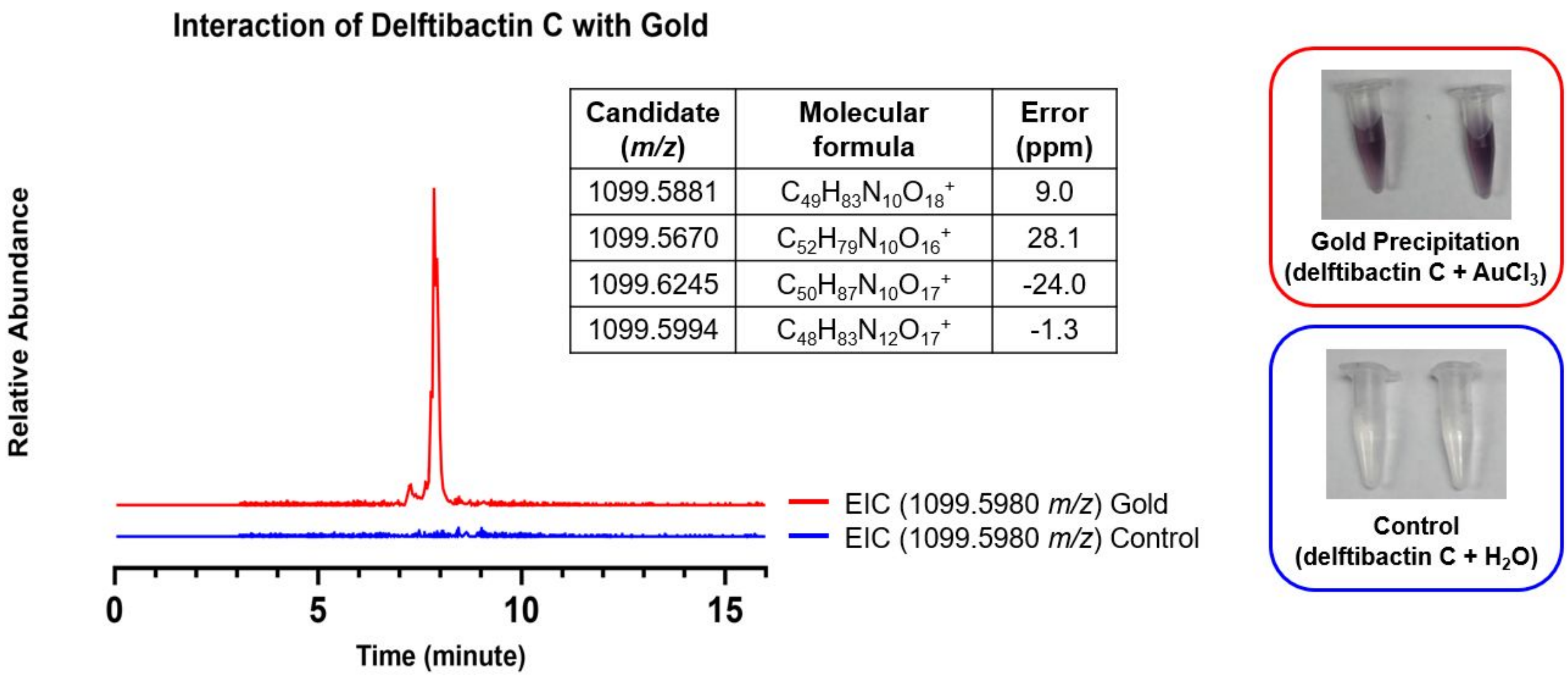

Candidate (*m/z*)	Molecular formula	Error (ppm)
1099.5881	$C_{49}H_{83}N_{10}O_{18}^{+}$	9.0
1099.5670	$C_{52}H_{79}N_{10}O_{16}^{+}$	28.1
1099.6245	$C_{50}H_{87}N_{10}O_{17}^{+}$	-24.0
1099.5994	$C_{48}H_{83}N_{12}O_{17}^{+}$	-1.3

The gold precipitate formed from gold chloride ($AuCl_3$), as reported by Johnston and coworkers,[1] was observed with delftibactin C (see **Red** and **Blue** boxes. Based on the observation of a mass at 1099.5950 *m/z* (**Red** chromatogram), we hypothesized the reaction led to an oxidative degradative product of delftibactin C but were unable to convincingly annotate the fragment spectrum of this mass (see SI Fig. S40).

Figure 40. Unannotated HRMS[2] Fragment Spectrum of 1099 *m/z* Product of Gold Reaction.

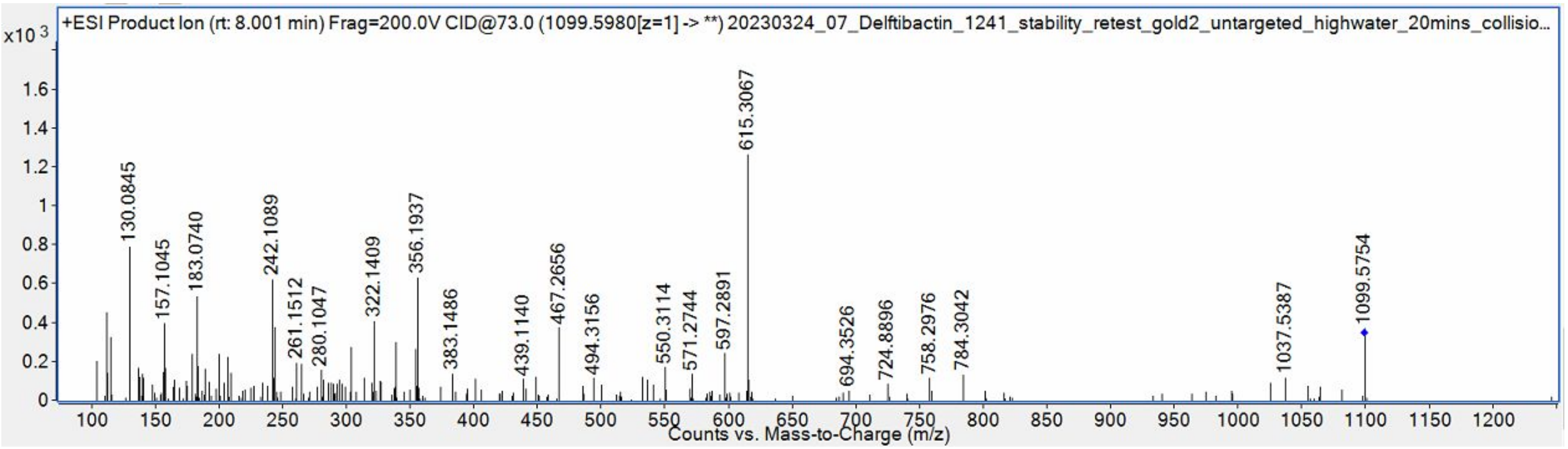

We hypothesized a set of highly speculative products of the reaction with the gold metal center (below), but these structures did not match convincingly with the observed spectrum for the 1099 *m/z* product in the gold reaction, which could not be annotated (above).

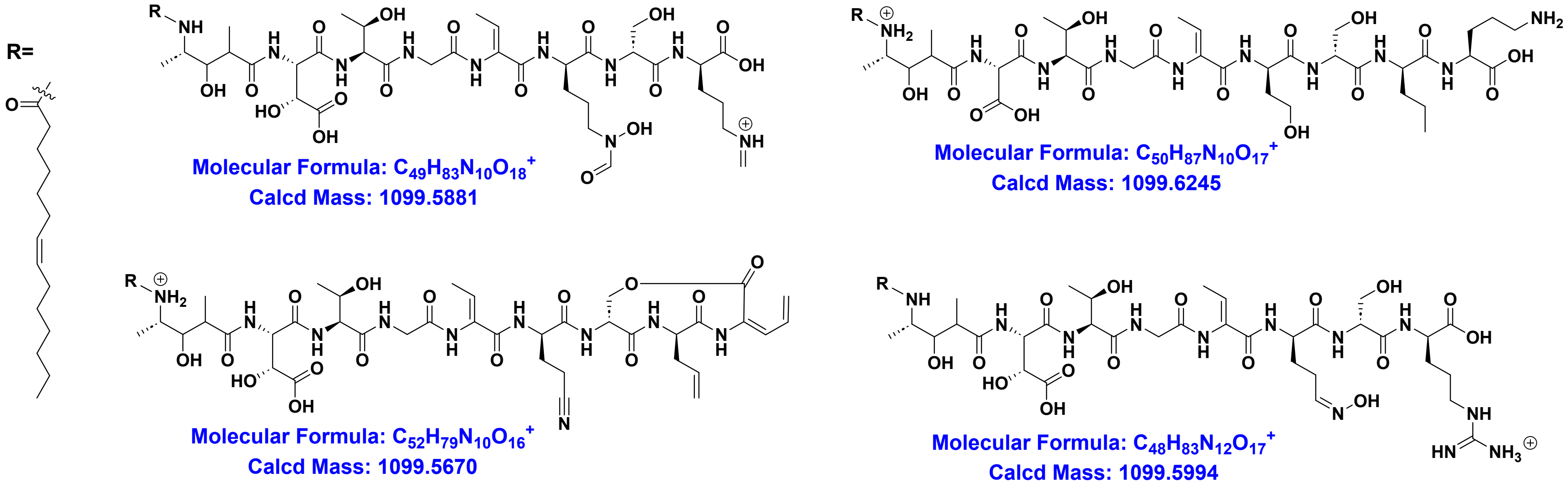

Table S2: MassQL Search for Cu-bound Masses after Copper Interaction with Delftibactin C (1)

MassQL Query	QUERY<br>MS1DATA<br>WHERE<br>MS1MZ=X-1.998:INTENSITYMATCH=Y*2.24:INTENSITYMATCHPERCENT=50:TOLERANCEPPM=10<br>AND<br>MS1MZ=X:INTENSITYMATCH=Y:INTENSITYMATCHREFERENCE:INTENSITYPERCENT=5<br>AND<br>MS1MZ=X+0.998:INTENSITYMATCH=Y*0.5:INTENSITYMATCHPERCENT=60<br>AND<br>MS1MZ=X-62.91214:TOLERANCEPPM=10<br>AND<br>MS2PREC=X<br>FILTER<br>MS1MZ=X
Plain Text Translation	Returning the scan information on MS1.<br>The following conditions are applied to find scans in the mass spec data.<br>Finding MS1 peak at m/z X-1.998 an expected relative intensity to reference peak of Y*2.24 and accepting variability of 50.0% in relative intensity and a 10.0 PPM tolerance.<br>Finding MS1 peak at m/z X an expected relative intensity to the reference peak of Y and this peak is used as the intensity reference for other peaks in the spectrum and a minimum percent intensity relative to a base peak of 5.0%.<br>Finding MS1 peak at m/z X+0.998 an expected relative intensity to a reference peak of Y*0.5 and accepting variability of 60.0% in relative intensity.<br>Finding MS1 peak at m/z X-62.91214 a 10.0 PPM tolerance.<br>Finding MS2 spectra with a precursor m/z X.<br>Finding MS1 peak at m/z X.
MassQL Query Link	http://proteomics2.ucsd.edu/ProteoSAFe/status.jsp?task=d975519654c447b18bede4615f2eda0e

References:

(1) Johnston, C. W.; Wyatt, M. A.; Li, X.; Ibrahim, A.; Shuster, J.; Southam, G.; Magarvey, N. A. Gold Biomineralization by a Metallophore from a Gold-Associated Microbe. *Nat. Chem. Biol.* **2013**, *9*, 241–243. https://doi.org/10.1038/nchembio.1179.

(2) Linne, U.; Doekel, S.; Marahiel, M. A. Portability of Epimerization Domain and Role of Peptidyl Carrier Protein on Epimerization Activity in Nonribosomal Peptide Synthetases. *Biochemistry* **2001**, *40* (51), 15824–15834. https://doi.org/10.1021/bi011595t.

(3) Reitz, Z. L.; Hardy, C. D.; Suk, J.; Bouvet, J.; Butler, A. Genomic Analysis of Siderophore β-Hydroxylases Reveals Divergent Stereocontrol and Expands the Condensation Domain Family. *Proc. Natl. Acad. Sci. U. S. A.* **2019**, *116* (40), 19805–19814. https://doi.org/10.1073/pnas.1903161116.

(4) Balibar, C. J.; Vaillancourt, F. H.; Walsh, C. T. Generation of D Amino Acid Residues in Assembly of Arthrofactin by Dual Condensation/Epimerization Domains. *Chem. Biol.* **2005**, *12* (11), 1189–1200. https://doi.org/10.1016/j.chembiol.2005.08.010.

(5) Scholz-Schroeder, B. K.; Soule, J. D.; Gross, D. C. The SypA, SypB, and SypC Synthetase Genes Encode Twenty-Two Modules Involved in the Nonribosomal Peptide Synthesis of Syringopeptin by Pseudomonas Syringae Pv. Syringae B301D. *Mol. Plant-Microbe Interact.* **2003**, *16* (4), 271–280. https://doi.org/10.1094/MPMI.2003.16.4.271.
